# T cell receptor signaling induces expression of lysine demethylase KDM6B to maintain Treg homeostasis

**DOI:** 10.1172/JCI196022

**Published:** 2026-04-23

**Authors:** Minghong He, Beisi Xu, Pria G. Bose, Morgan J. McCullough, Rani S. Sellers, Xinying Zong, Wenjie Qi, Brianna L. Banten, Miriya K. Tune, Matthew P. Zimmerman, Genevieve Mullins, Brian C. Miller, J. Justin Milner, Jason K. Whitmire, Ageliki Tsagaratou, Karl B. Shpargel, Claire M. Doerschuk, Yong-Dong Wang, Jacob A. Steele, Shondra M. Pruett-Miller, Yongqiang Feng, Jason R. Mock

**Affiliations:** 1 Department of Immunology, and; 2 Center for Applied Bioinformatics, St. Jude Children’s Research Hospital, Memphis, Tennessee, USA.; 3 Marsico Lung Institute,; 4 Department of Pathology and Laboratory Medicine,; 5 Department of Microbiology and Immunology,; 6 Department of Medicine, Division of Pulmonary Diseases and Critical Care Medicine,; 7 Department of Cell Biology and Physiology, and; 8 Lineberger Comprehensive Cancer Center, University of North Carolina, Chapel Hill, North Carolina, USA.; 9 Department of Medicine, Division of Oncology, and; 10 Department of Genetics, University of North Carolina at Chapel Hill, Chapel Hill, North Carolina, USA.; 11 Center for Airways Disease, School of Medicine, University of North Carolina, Chapel Hill, North Carolina, USA.; 12 Department of Cell and Molecular Biology, and; 13 Department of Tumor Cell Biology, St. Jude Children’s Research Hospital, Memphis, Tennessee.

**Keywords:** Immunology, Pulmonology, Cellular immune response, Epigenetics, Tregs

## Abstract

Tregs expressing forkhead box P3 (FOXP3) play crucial roles in maintaining immune tolerance and tissue integrity. EZH2, a histone H3 lysine 27 (H3K27) methyltransferase, is known as a key regulator of Treg identity and suppressive function upon activation. Here, we demonstrate that the H3K27 lysine demethylase KDM6B, which catalyzes the opposing reaction to EZH2, is also required for Treg identity and function after activation. Treg-specific deletion of *Kdm6b* impaired tissue Treg fate and function. KDM6B was upregulated after T cell antigen receptor signaling in Tregs and contributed to the regulation of Treg-associated gene expression through both direct and indirect mechanisms. A subset of Treg functional genes were direct targets of KDM6B and were co-occupied by FOXP3 at *cis*-regulatory regions, where KDM6B recruitment limited H3K27me3 accumulation. More broadly, KDM6B-dependent H3K27 demethylation facilitated Treg gene expression programs that supported tissue Treg homeostasis.

## Abstract 2

Kdm6b supports regulatory T cells in maintaining their role and stability in tissues during inflammation, injury, or aging.

## Graphical abstract

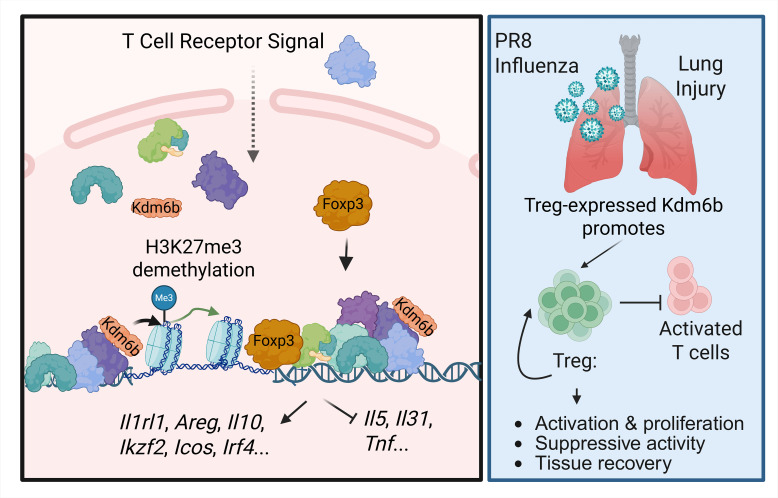

## Introduction

FOXP3^+^ Tregs have immunosuppressive and tissue-reparative functions during resolution of tissue injuries. Tregs are identified by expression of the X-linked nuclear protein forkhead box protein 3 (FOXP3) (1–3) and play crucial roles in immunosuppression and tissue repair (4–7). Treg immunosuppressive effects occur through multiple mechanisms, including expression of surface molecules and soluble mediators (4, 8–12). Furthermore, Tregs interact with and regulate the functions of innate immune cells, including dendritic cells, γδ T cells, macrophages, and neutrophils (13), and they promote tissue repair (5, 6). Treg pleiotropic functions underline their pivotal role in the immune system.

Tregs increase in number during resolution in several experimental models, including lung injury (14). These tissue Tregs are present in the lungs of patients with acute respiratory distress syndrome, suggesting that they may be an important determinant in the resolution of that condition (7, 15). Published studies of murine acute lung injury (ALI) models showed that during the resolution phase of injury, Tregs increase in abundance and undergo distinct transcriptional reprogramming, compared with Tregs from uninjured control lungs (7, 16).

Lysine-specific demethylase 6B (*Kdm6b*) (also referred to as Jumonji domain-containing protein-3 [*Jmjd3*]) was differentially regulated in resolving lung Tregs in an LPS-induced experimental lung injury model compared with steady-state lung Tregs (14). KDM6B removes methyl groups from histone 3 lysine 27 (H3K27) tails, critically influencing chromatin organization and epigenetic regulation of gene expression (17, 18). Trimethylation of H3K27 (H3K27me3) contributes to gene repression by recruiting Polycomb complexes, such as Polycomb-repressive complex 1 (19). Demethylation of H3K27me3 is associated with a more open chromatin state and gene activation, and H3K27 methylation has been reported to influence transcriptional changes that regulate immune cell development, T cell trafficking, and T cell responses (19–22). The methylation of H3K27 is mediated by Polycomb-repressive complex 2, the catalytic subunit of which is enhancer of zeste 2 polycomb repressive complex 2 subunit (EZH2) (19, 22, 23). EZH2 plays an important role in lymphocyte development and differentiation (24, 25). Studies have reported that Ezh2 deficiency in Tregs impairs their fate and function (22, 26). Therefore, given that H3K27 methylation status is important in immune cell function and that the specific function of KDM6B in Tregs are not well understood, we sought to examine the effects of *Kdm6b* deficiency in Tregs at steady state and during the resolution of lung injury.

In this study, we demonstrated that KDM6B is induced by TCR signaling and subsequently deposited onto FOXP3-bound chromatin. To investigate the role of KDM6B in Treg function, we generated *Kdm6b^ΔTreg^* mice to deplete KDM6B in Tregs. In an inflammatory lung injury model, we found that KDM6B-deficient Tregs fail to expand in number during ALI resolution. Additionally, *Kdm6b*-deficient Tregs have altered H3K27me3 methylation states and transcriptional profiles, highlighting KDM6B’s role in maintaining Treg identity and function.

These findings and those of prior studies (22, 26) suggest a coordinated balance of H3K27 methylation states in Tregs upon TCR signaling, which are important for directing their functions. Dysregulation of either H3K27 methyltransferase activity (i.e., EZH2), as previously shown (23, 26), or H3K27 demethylase activity (KDM6B), as shown in this study, led to alterations in gene transcription that contributed to suboptimal Treg function (22, 26). Therefore, the balance between methyltransferase and demethylase activity on H3K27 methylation states in Tregs is essential for maintaining optimal Treg-directed immune homeostasis and tolerance upon antigen stimulation.

## Results

### TCR signaling induced KDM6B is deposited onto FOXP3-bound chromatin to promote Treg homeostasis.

**Figure 1:**
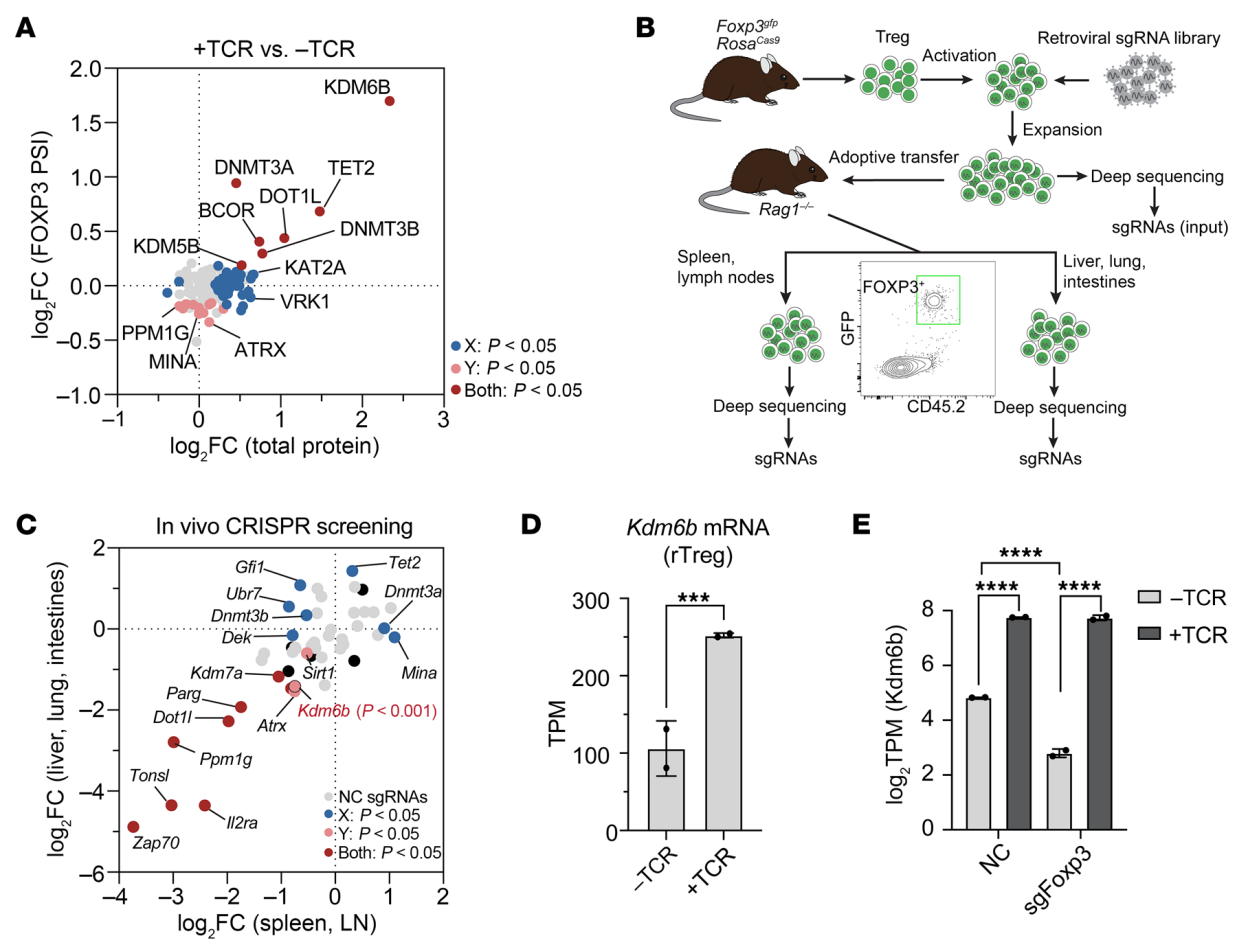
KDM6B is enriched in proximity to FOXP3 in response to TCR signaling and required for in vivo Treg fitness. (**A**) Comparison of total (*x* axis) versus FOXP3-proximal (*y* axis) levels of epigenetic regulators in induced Tregs with or without TCR stimulation (anti-CD3/CD28) (28). (**B**) Schematic of in vivo CRISPR screen. A retroviral sgRNA library targeting 21 FOXP3-proximal epigenetic regulators whose proteins were differentially represented in proximity to FOXP3 (in **A**), plus *Zap70* and *Il2ra*, was transduced into Tregs from *Foxp3^gfp^ Rosa^Cas9^* mice. After 3–4 days of in vitro expansion, these CD45.2^+^ cells were sorted, and GFP^+^ Tregs were cotransferred with total CD45.1^+^ CD4^+^ T cells, including Tregs, into *Rag1^–/–^* mice. Then, 2 weeks later, cells were recovered from spleens, lymph nodes, liver, lungs, and small and large intestines. CD45.2^+^GFP^+^ Tregs were sorted to assess sgRNA representation by high-throughput sequencing. (**C**) Fold changes (FCs) of sgRNAs targeting genes identified by FOXP3 PSI in Tregs recovered from lymphoid (spleen and lymph nodes) and nonlymphoid organs (liver, lung, small and large intestines) after CRISPR deletion and adaptation in *Rag1^–/–^* mice, as described in **B**. Data are summarized from 4 biological replicates. Read counts of 4 sgRNAs targeting the same gene were combined for a paired Student’s *t* test by comparing with NCs. Log2 FCs were determined by comparing recovered cells and inputs. (**D**) *Kdm6b* expression in induced Tregs was quantified by RNA-Seq under conditions with or without TCR stimulation. Data are reported as the mean ± SD (*n =* 2); DESeq2 with Benjamini-Hochberg correction (***q < 0.001). (**E**) Log_2_(TPM) *Kdm6b* expression (RNA-Seq) in control and *Foxp3* CRISPR-deleted rTregs with or without TCR restimulation. DESeq2 with Benjamini-Hochberg correction (****q < 0.0001). TPM, transcripts per million RNA molecules.

Given that FOXP3-interacting proteins play crucial roles in regulating Treg fate and immunosuppressive function (27–29), we used a proteomics approach to investigate proteins in proximity to FOXP3. This approach, known as projection of spatial information (PSI), was followed by mass spectrometry–based protein identification (28). Using Tregs induced in vitro (iTregs) that model the key characteristics of Tregs isolated ex vivo (30), we observed the recruitment and depletion of proteins in proximity to FOXP3 after TCR signaling (28). Among these, 197 detected epigenetic regulators were further explored, encompassing chromatin remodeling and DNA and histone modifications. Many of these epigenetic regulators exhibited significant alterations; for instance, KDM6B, TET2, DOT1I, DNMT3A/B, BCOR, and KDM5B showed substantial (*P* < 0.05) increases in both total protein levels and FOXP3 proximal enrichment, whereas PPM1G, MINA, and ATRX demonstrated decreased FOXP3 proximal enrichment without significant alterations in total protein quantities (Figure 1A).

To determine how these dynamic chromatin proteins may play a role in Tregs, we synthesized a retroviral single-guide RNA (sgRNA) library targeting 21 epigenetic regulators differentially represented by FOXP3 PSI upon TCR and costimulation (Supplemental Tables 1 and 2; supplemental material available online with this article; https://doi.org/10.1172/JCI196022DS1). Guide RNAs targeting *Il2ra* and *Zap70*, which are crucial for Treg function and TCR signal transduction, respectively, were included in the library as controls. We transduced these sgRNAs into Tregs isolated from *Foxp3^gfp^ Rosa^Cas9^* mice (28) to examine the effects of their CRISPR-mediated deletions on Treg survival or fitness after adoptive transfer into *Rag1^–/–^* mice (Figure 1B). In the presence of congenically marked WT total CD4^+^ T cells, the frequencies of sgRNAs in Tregs (GFP^+^) from lymphoid organs (spleen and lymph nodes) and nonlymphoid organs (liver, lung, and intestines) were compared with those in the input cells 2 weeks later. We pooled the sgRNAs against the same genes and normalized their reads with the total reads of nontargeting (negative control [NC]) sgRNAs. This stringent analysis identified several genes as pivotal regulators of Treg survival or fitness, including Kdm6b (Figure 1C).

*Kdm6b* expression was markedly increased upon TCR stimulation in ex vivo isolated resting Tregs (rTregs) (Figure 1D), in line with the total protein levels in iTregs (Figure 1A). This increase was not affected by *Foxp3* deletion (Figure 1E). These findings collectively suggest that TCR signaling–induced *Kdm6b* may play a pivotal role in Tregs, likely by collaborating with FOXP3 in transcriptional regulation of functional genes upon activation.

Additionally, prior work demonstrated that perturbing H3K27 methyltransferase activity by targeting EZH2 disrupts gene transcription and impairs Treg function downstream of CD28 signaling (26). Because of KDM6B’s potential complementary role in shaping the H3K27 landscape in Tregs, it was selected as a key target for further investigation. Notably, in this earlier study, KDM6B was not identified as a histone lysine demethylase that is significantly regulated by CD28 signaling (26).

### Conditional deletion of Kdm6b in Tregs leads to moderate autoimmune activation.

**Figure 2:**
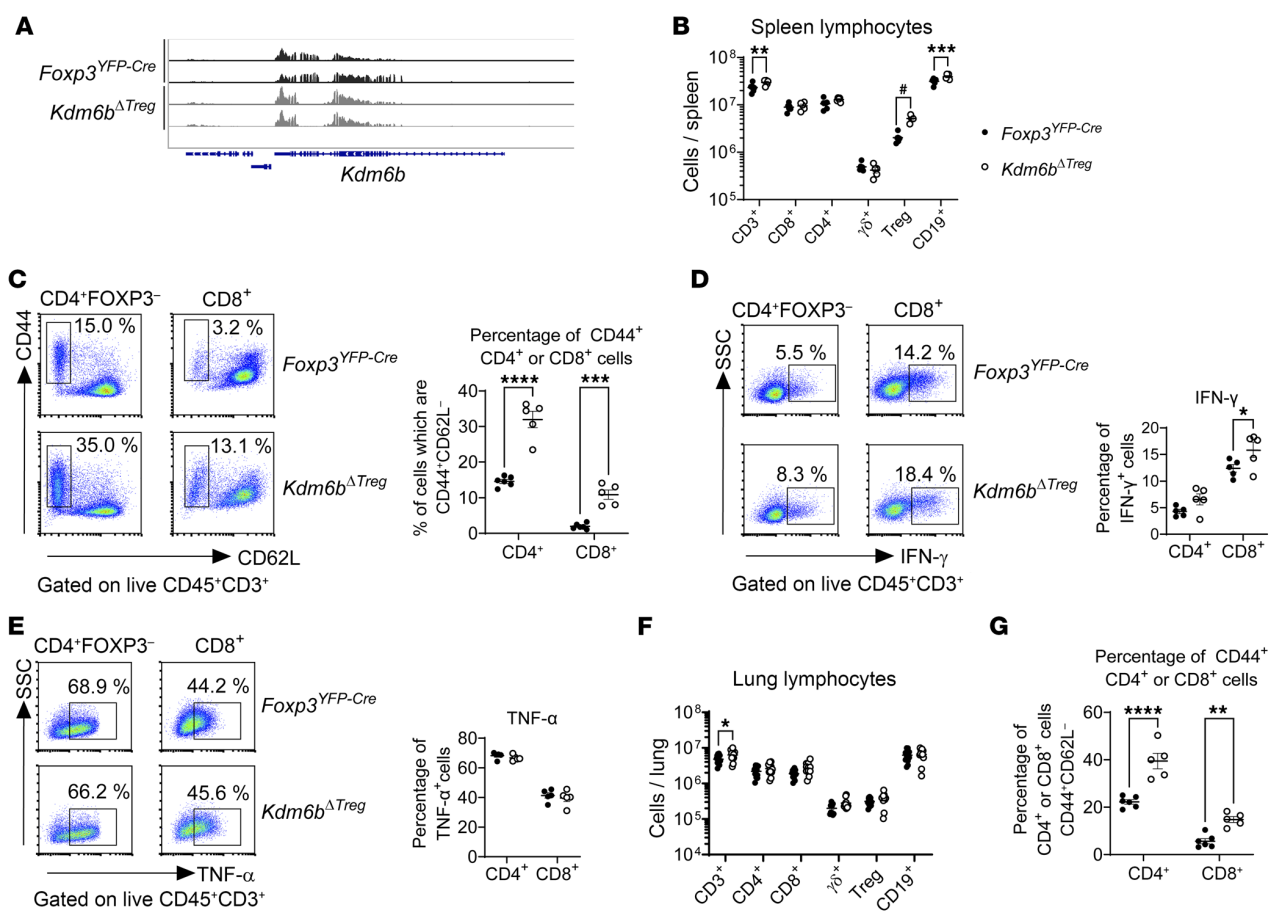
Conditional deletion of *Kdm6b* in Tregs leads to moderate autoimmune activation. (**A**) Comparison of *Kdm6b* expression mapped across exons from sorted CD4^+^FOXP3^+^CD62L^hi^CD44^low^ rTregs in either *Foxp3^YFP-Cre^* or *Kdm6b^ΔTreg^* mice determined by RNA-Seq. Exons 14–20 of the 24 total exons are deleted in *Kdm6b^ΔTreg^* mice. (**B**–**E**) Male and female spleen single-cell suspensions from either *Foxp3^YFP-Cre^* or *Kdm6b^ΔTreg^* mice, aged 8–12 weeks, were used in flow cytometric analysis of cells at steady state. (**B**) Lymphocyte immunophenotyping in splenocytes was similar to that previously described (14). (**C**) Frequency of CD4^+^FOXP3^-^ or CD8^+^ T cells that are CD44^+^CD62L^–^ in *Foxp3^YFP-Cre^* or *Kdm6b^ΔTreg^* splenocytes. (**D** and **E**) Splenocytes were stimulated with PMA and ionomycin in the presence of brefeldin for 6 hours and examined for cytokine expression in CD4^+^ and CD8^+^ T cells for (**D**) IFN-γ or (**E**) TNF-α (*n =* 5–6 mice per strain; representative of 2 separate experiments). (**F** and **G**) Male and female lung single-cell suspensions from either *Foxp3^YFP-Cre^* or *Kdm6b^ΔTreg^* mice, aged 8–12 weeks, were used in flow cytometric analysis of cells at steady state. (**F**) Lymphocyte immunophenotyping (*n =* 11–12 mice per strain; a combination of 2 separate experiments). (**G**) Frequency of CD4^+^Foxp3^–^ or CD8^+^ T cells that are CD44^+^CD62L^–^ in *Foxp3*^YFP-Cre^ or *Kdm6b^ΔTreg^* lung lymphocytes (*n =* 5–6 mice per strain; representative of 2 separate experiments). Data are presented as the mean ± SEM. *P* values were derived from unpaired 2-tailed *t* tests (^#^*P* < 0.05) (**B**) or 2-way ANOVA with Holm-Šidák multiple comparisons (**B**–**G**): *P < 0.05, **P < 0.01, ***P < 0.001, ****P < 0.0001.

To determine whether KDM6B influences Treg function, we bred *Kdm6b^fl/fl^* mice (31) with *Foxp3^YFP-Cre^* mice (9) to obtain a strain (*Kdm6b^ΔTreg^*) in which Tregs do not express *Kdm6b*. RNA-Seq confirmed the deletion of *Kdm6b* exons 14-20 in *Kdm6b^ΔTreg^* mice (Figure 2A).

Immunophenotyping splenic cells, as previously described (14), demonstrated more total CD3^+^ T and CD19^+^ B lymphocytes in *Kdm6b^ΔTreg^* mice than in *Foxp3^YFP-Cre^* controls (Figure 2B). Although Treg number did not reach statistical significance by multiple comparisons analysis, there was an approximately 2.5-fold increase in splenic Treg number in *Kdm6b^ΔTreg^* mice when assessed by an unpaired *t* test (Figure 2B). Furthermore, the proportions of activated CD44^hi^CD62L^lo^ CD4^+^ or CD44^hi^CD62L^lo^ CD8^+^ conventional T cells were markedly increased in splenocytes from *Kdm6b^ΔTreg^* mice (Figure 2C). IFN-γ expression was greater in CD8^+^ T splenocytes from *Kdm6b^ΔTreg^* mice after stimulation with phorbol 12-myristate 13-acetate (PMA)/Ionomycin, whereas there was no change in TNF-α expression after stimulation in either CD4^+^ or CD8^+^ splenocytes from either strain (Figure 2, D and E).

A prior study found reduced Kdm6b expression in lung Tregs during early resolution after LPS-induced injury compared with uninjured lungs, suggesting a role for KDM6B in lung Treg function (14). To assess whether *Kdm6b* gene knockout disrupts lung Treg function, we enumerated total lung cell numbers from enzymatically digested lung single-cell suspensions. The number of CD3^+^ T cells was greater in *Kdm6b^ΔTreg^* mice (Figure 2F), as was the proportion of activated CD44^hi^CD62L^lo^ CD4^+^ T cells and CD44^hi^CD62L^lo^ CD8^+^ T cells in the lung (Figure 2G). Native-state histology of organs supported the immunophenotyping results, showing that *Kdm6b* deletion in Tregs led to mild microscopic changes, most noticeable as increased germinal center activation in the spleen (Supplemental Figure 1). Thus, *Kdm6b* deficiency in Tregs is associated with increased basal levels of T cell activation compared with that in *Foxp3^YFP-Cre^* control mice.

### Kdm6b deletion impairs Treg function.

**Figure 3:**
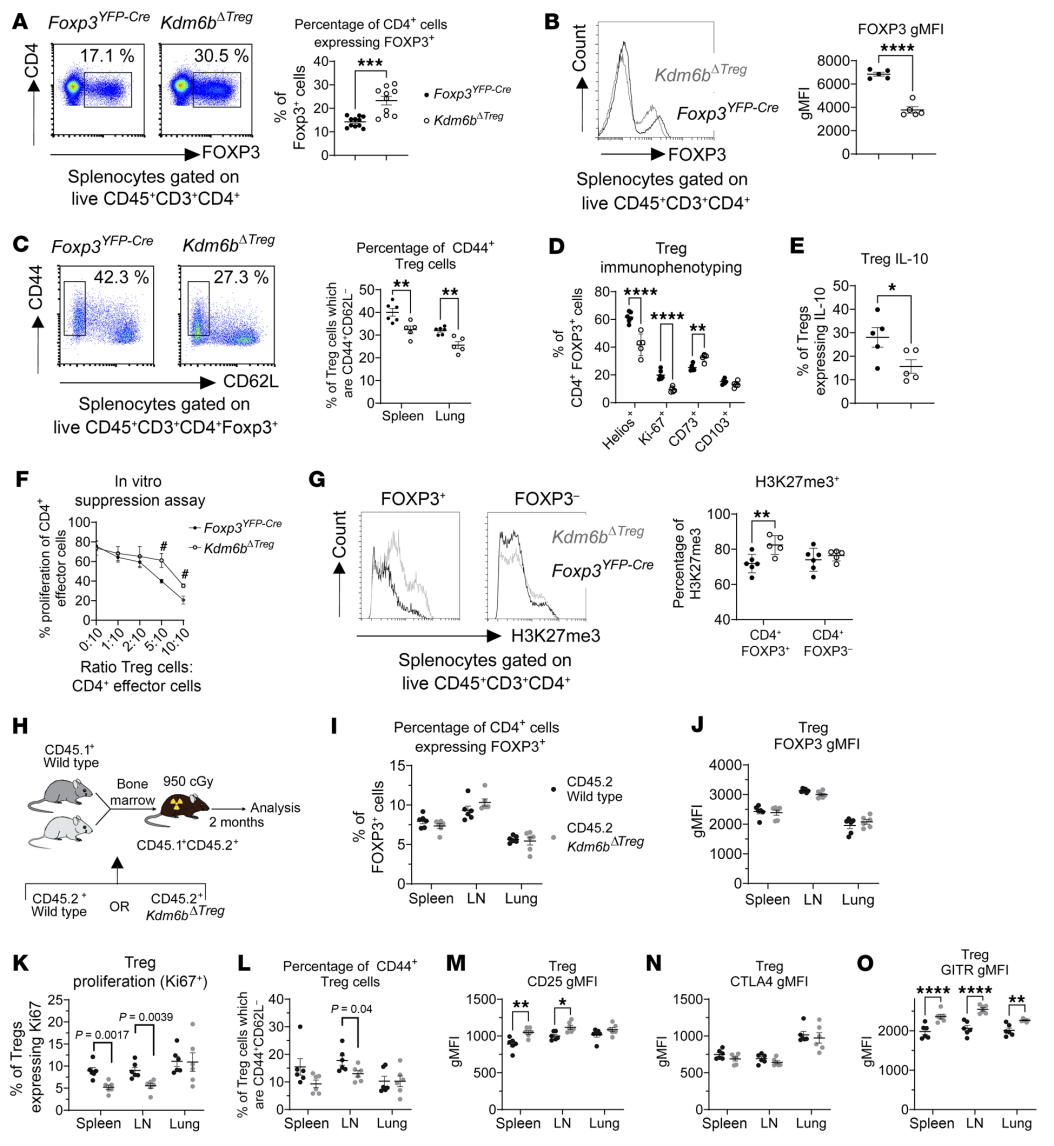
Kdm6b deletion impairs Treg function. Single-cell suspensions from spleens of male and female *Foxp3*^YFP-Cre^ or *Kdm6b^ΔTreg^* mice (aged 8–12 weeks) were analyzed by flow cytometry at steady state. (**A**) Frequency of FOXP3^+^ Tregs among CD4^+^ lymphocytes in spleen (*n =* 10–11 mice/strain, combining 2 separate experiments). (**B**) FOXP3 expression measured as gMFI (*n =* 5 mice/strain, representative of 2 separate experiments). (**C**) Frequency of CD44^+^CD62L^–^ Tregs in splenic CD4^+^FOXP3^+^ cells (*n =* 5–6 mice/strain, representative of 2 separate experiments). (**D**) Immunophenotyping of splenic CD4^+^FOXP3^+^ lymphocytes (*n =* 5–6 mice/strain, representative of 2 separate experiments). (**E**) IL-10 production by splenic Tregs after PMA/ionomycin stimulation (*n =* 5 mice/strain, representative of 2 separate experiments). (**F**) In vitro suppression of CD4^+^ effector T cell proliferation by splenic Tregs (combined data from 3 independent assays). ^#^*P* < 0.05. (**G**) H3K27me3 abundance in CD4^+^FOXP3^+^ or CD4^+^FOXP3^–^ splenocytes (*n =* 5–6 mice/strain). (**H**) Experimental schematic of mixed bone-marrow chimeras (*n =* 6 mice/condition). (**I**–**L**) Analysis of CD45.2^+^ donor-derived cells showing Treg frequency (**I**), FOXP3 expression (**J**), Ki67^+^ proliferation (**K**), and CD44^+^CD62L^–^ effector phenotype (**L**) in spleen, lymph nodes (LN), and lung. (**M**–**O**) Expression (gMFI) of CD25 (**M**), CTLA4 (**N**), and glucocorticoid-induced TNFR-related protein (GITR) (**O**) on donor-derived Tregs across tissues. Data are given as the mean ± SEM. Statistical significance was determined by unpaired 2-tailed *t* tests or 2-way ANOVA with Holm-Šídák correction, as indicated. *P < 0.05, **P < 0.01, ***P < 0.001, ****P < 0.0001.

To assess the function of Tregs with *Kdm6b* deficiency, Tregs obtained from either the spleen or lung single-cell suspensions were analyzed. The percentage of FOXP3^+^ Tregs among CD4^+^ lymphocytes was greater in spleens (Figure 3A), and FOXP3 expression measured by median fluorescence intensity (MFI) was less in *Kdm6b^ΔTreg^* mice (Figure 3B). Tregs obtained from either splenocytes or lung single-cell suspension had a lower proportion of activated CD44^hi^CD62L^lo^ Tregs in *Kdm6b^ΔTreg^* mice compared with *Foxp3^YFP-Cre^* control mice (Figure 3C). Further immunophenotypic analysis demonstrated a high percentage of CD73^hi^ expression in the CD4^+^FOXP3^+^ Treg population and lower percentages of Helios (IKZF2) and Ki-67 positivity in CD4^+^FOXP3^+^ Tregs in *Kdm6b^ΔTreg^* mice (Figure 3D).

CD73-dependent adenosine generation has been shown to promote lung injury resolution, whereas Helios contributes to stability of Treg lineage (32, 33). Splenic Tregs were stimulated with PMA and ionomycin in the presence of brefeldin to examine cytokine expression. IL-10 cytokine expression was decreased in *Kdm6b^ΔTreg^* Tregs compared with the control (Figure 3E). This finding correlated with data from an in vitro effector CD4^+^ cell suppression assay, which demonstrated a lower suppressive capacity of CD4^+^FOXP3^+^ Tregs deficient in KDM6B compared with Tregs from *Foxp3^YFP-Cre^* mice (Figure 3F).

As an H3K27me3 demethylase, KDM6B catalyzes the removal of H3K27me3 modification. Indeed, flow cytometric analysis of splenic CD4^+^FOXP3^+^ Tregs lacking KDM6B showed a significant increase in H3K27me3 compared with WT Tregs (*Foxp3^YFP-Cre^* mice) (Figure 3G). In contrast, no difference in H3K27me3 was detected in CD4^+^FOXP3^–^ lymphocytes, demonstrating a Treg-specific effect in the *Kdm6b^ΔTreg^* mice (Figure 3G).

Mixed bone-marrow chimeric mice were used to investigate potential compensatory effects on these findings in *Kdm6b^ΔTreg^* mice (Figure 3H). Within this competitive setting, the percentage of FOXP3^+^ cells among CD4^+^ T cells was comparable between WT and *Kdm6b*-deficient Tregs across spleen, lymph node, and lung compartments (Figure 3I). Similarly, FOXP3 protein expression, assessed by geometric MFI (gMFI), was not significantly altered in *Kdm6b*-deficient Tregs relative to controls (Figure 3J). However, despite similar representation and FOXP3 expression, *Kdm6b*-deficient Tregs displayed evidence of cell-intrinsic effects of *Kdm6b* deletion on Tregs, including a lower proportion of CD44^+^ and proliferating Tregs marked by Ki-67 expression (Figure 3, K and L).

Phenotypic analysis of key suppressive and activation-associated markers further revealed selective changes in *Kdm6b*-deficient Tregs. CD25 expression was modestly increased, whereas CTLA-4 expression was largely preserved across tissues (Figure 3, M and N). In contrast, glucocorticoid-induced TNFR-related protein expression was significantly elevated in *Kdm6b*-deficient Tregs in all compartments examined (Figure 3O).

We additionally evaluated the percentages of thymic Tregs among CD4^+^ T cells and their activation state (Supplemental Figure 2). In the thymus, the percentages of Tregs (FOXP3^+^) in the CD4^+^ population did not differ between WT and *Kdm6b*-deficient compartments. However, there were decreased percentages of CD44^hi^CD62L^lo^ Tregs in *Kdm6b^ΔTreg^* mice compared with WT controls, with no difference in CD44^hi^CD62L^lo^ CD4^+^ FOXP3^–^ cells. Interestingly the percentage of FOXP3+CD25- Treg cells in the thymus of *Kdm6b^ΔTreg^* mice was decreased, indicating shifted Treg subpopulations after induction (Supplemental Figure 2). Thus, KDM6B fine-tunes steady-state Treg homeostasis and function.

### KDM6B coordinates with FOXP3 to regulate Treg fate.

**Figure 4:**
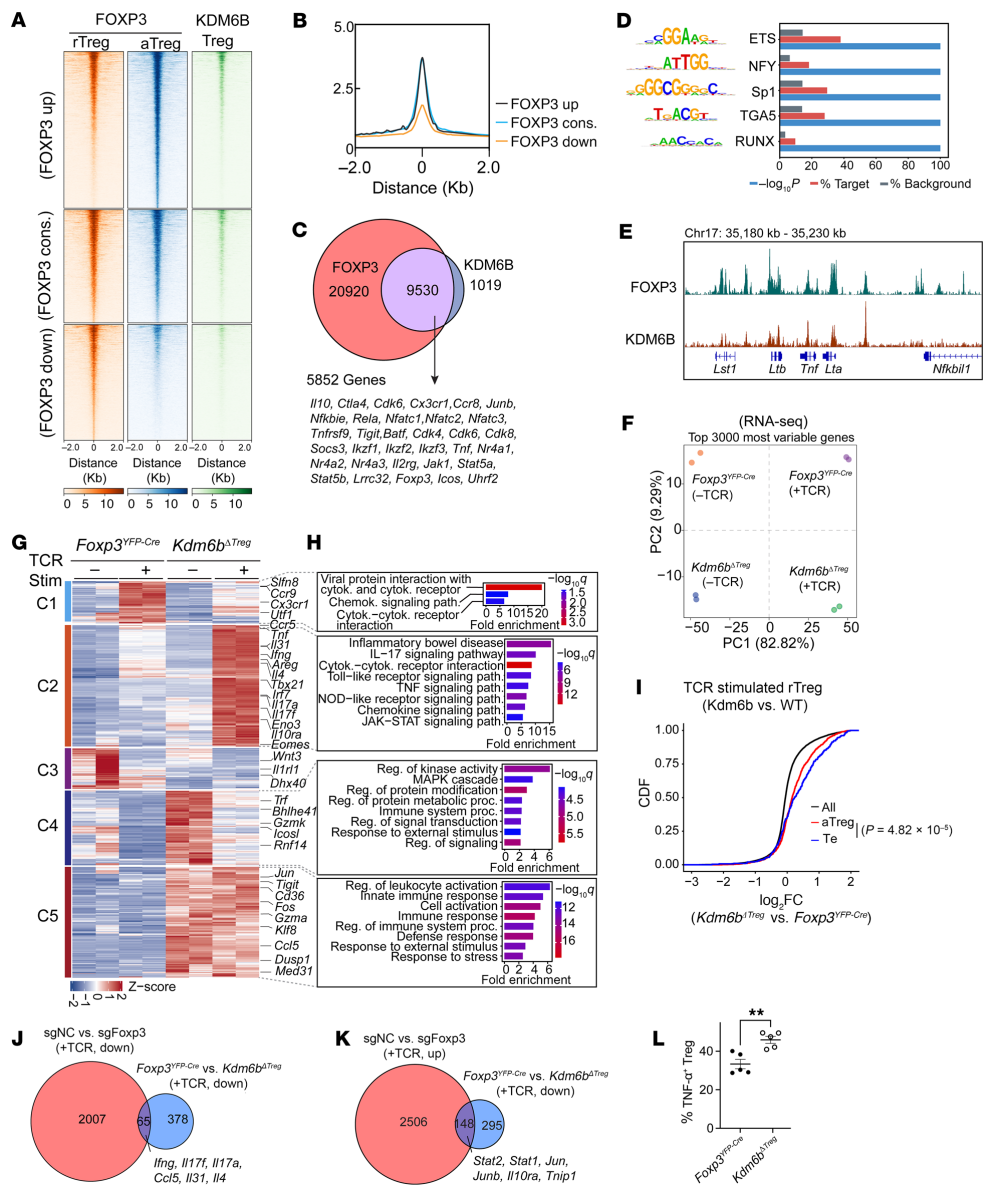
KDM6B coordinates with FOXP3 to regulate Treg functional genes. (**A**) A heatmap of FOXP3 and KDM6B CUT&RUN showing binding at 3 different FOXP3 binding regions in aTreg and rTregs: increased (Up) (*P* < 0.05), constitutive (Cons) (*P* > 0.5; 0.95 < fold change [FC] < 1.05), and decreased (Down) (*P* < 0.05). Two replicates per condition were combined for analysis. FOXP3 CUT&RUN data were adapted from He et al. (28). (**B**) Average density signal of KDM6B binding at the 3 different FOXP3 binding regions in **A**. (**C**) Number of peaks (top) and genes (bottom) linked to the overlapped regions between FOXP3 (all reproducible peaks) and KDM6B binding peaks. (**D**) DNA sequence motifs of transcription factors enriched at KDM6B binding sites. (**E**) FOXP3 and KDM6B binding peaks at the *Tnf* gene region. (**F**) Principal component (PC) analysis of RNA-Seq results. RNA-Seq was performed in rTregs from lymphoid organs of *Foxp3^YFP-Cre^* or *Kdm6b^ΔTreg^* mice stimulated with plate-bound anti-CD3 and anti-CD28 antibodies (TCR) for 3 hours. Two replicates per condition were combined for analysis. (**G**) A heatmap showing expression patterns of genes in rTregs indicated in **F**. (**H**) Functional annotation of genes in clusters C1–C4. (**I**) Effect of *Kdm6b* deficiency on the expression of genes linked to aTreg program or effector T cell program. (**J** and **K**) Number of genes regulated by FOXP3 (sgNC- versus sgFoxp3-transduced Tregs) (28) and KDM6B. (**L**) Frequency of TNF-α expression Tregs in the spleen of *Foxp3^YFP-Cre^* or *Kdm6b^ΔTreg^* mice (*n =* 5 mice/strain, representative of 2 separate experiments). **P < 0.01 by 2-tailed *t* test. CDF, cumulative distribution function; Chemok, chemokine; cytok, cytokine; Reg, regulation.

Given the spatial proximity of FOXP3 and KDM6B (Figure 1A), we set out to determine if they coordinate to regulate the expression of Treg functional genes. FOXP3 CUT&RUN (Cleavage Under Targets & Release Using Nuclease) revealed substantial overlap (90%) between KDM6B peaks and our published FOXP3 peaks (28) in both activated Tregs (aTregs) and rTregs (Figure 4, A–C). Consistent with the spatial proximity data of FOXP3, KDM6B primarily occupied regions with increased or constitutive FOXP3 binding in aTregs compared with rTregs (Figure 4, A and B). These regions were enriched with transcription factor motifs, including ETS, NFY, SP1, TGA5, and RUNX (Figure 4D), some of which are known important regulators of Treg differentiation and function. Both FOXP3 and KDM6B bind to crucial Treg functional genes, such as *Il10*, *Ctla4*, *Cx3cr1*, and *Tnf* (Figure 4, C and E).

To assess the effect of *Kdm6b* deficiency on gene expression during Treg activation, rTregs were sorted from WT and *Kdm6b^ΔTreg^* mice to perform RNA-Seq with or without 3 hours of stimulation by TCR agonists. This revealed significant effects of *Kdm6b^ΔTreg^* before and after stimulation, which could be categorized into at least 5 distinct clusters (Figure 4, G and H, and Supplemental Tables 3 and 4). Differentially expressed genes (DEGs), such as those in clusters C1–C4, are involved in many processes, including immune regulation as well as Treg differentiation and function (Figure 4H). Importantly, several proinflammatory cytokine genes were significantly upregulated in *Kdm6b^ΔTreg^* Tregs upon stimulation (C2), indicating a crucial role of Kdm6b in suppressing their expression. This notion was supported by the enrichment of effector T cell signature genes compared with aTreg signature genes selected by an unbiased approach (Figure 4I). We assessed *Ezh2* transcription with and without TCR stimulation from WT and *Kdm6b^ΔTreg^* mice, which demonstrated increased *Ezh2* expression in Kdm6b-deficient Tregs after TCR stimulation relative to WT controls (Supplemental Table 4).

The upregulation of cytokine genes in *Kdm6b^ΔTreg^* Tregs was comparable to the effects of *Foxp3* deletion (28), although the latter exhibited a more pronounced impact, as expected (Figure 4, J and K). Increased transcripts of cytokine genes were mostly beyond detection by flow cytometry, except for TNF-α (Figure 4L). KDM6B and FOXP3 also have distinct and independent roles in Tregs, and the pattern of peak overlap further highlights that FOXP3 exerts additional functions beyond those mediated by KDM6B (Figure 4, J and K). Nevertheless, TCR signaling induces KDM6B to extensively coordinate with FOXP3 to maintain Treg fate and function, in addition to other FOXP3-independent roles.

### KDM6B regulates chromatin accessibility and transcriptional programs in Tregs after TCR stimulation.

As expected, loss of *Kdm6b* led to a global increase in H3K27me3 enrichment at numerous loci (Figure 3G), consistent with its role as a histone demethylase. However, RNA-Seq analysis revealed that many genes were upregulated in Kdm6b-deficient Tregs compared with controls, both at steady state and after TCR stimulation.

**Figure 5:**
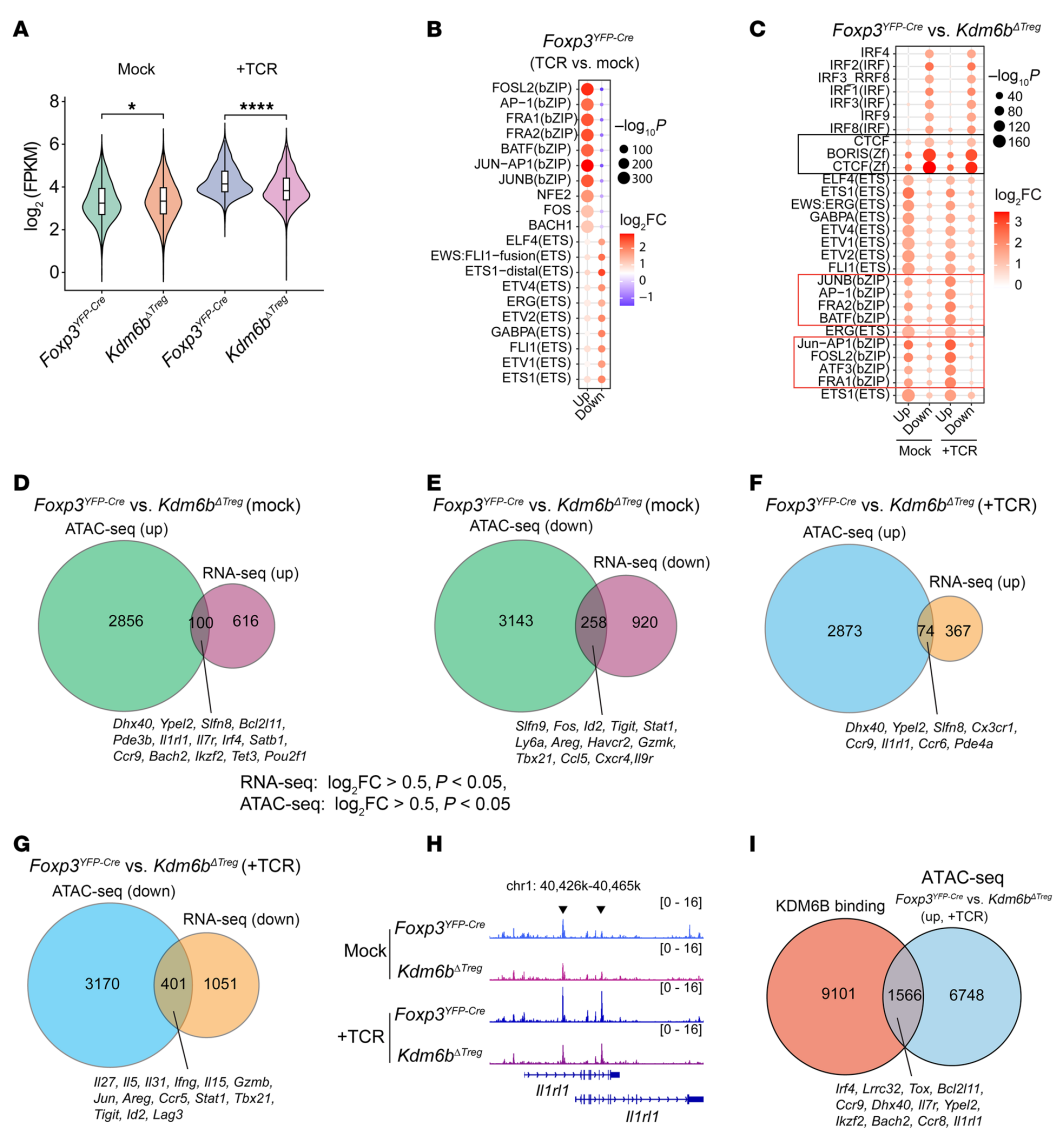
KDM6B regulates chromatin accessibility and transcriptional programs in Tregs after TCR stimulation. (**A**) Comparison of ATAC-Seq signal (FPKM) at genomic regions with differential accessibility in TCR- versus mock-treated WT Tregs (*P* < 0.05; log_2_FC > 0.5). *P < 0.05 and ****P < 0.0001, by unpaired 2-tailed *t* test. Three replicates were combined for analysis. (**B** and **C**) Transcription factor binding motif enrichment analysis of ATAC-Seq peaks under the indicated conditions. (**D**–**G**) Integration of ATAC-Seq and RNA-Seq datasets showing overlap between differentially accessible chromatin regions and DEGs under the indicated conditions. Three replicates were combined for analysis. (**H**) ATAC-Seq modification tracks for *Il1rl1* (2 alternative transcripts shown) comparing *Foxp3^YFP-Cre^* or *Kdm6b^ΔTreg^* splenic Tregs in the presence or absence of TCR stimulation. Data were pooled from 3 replicates for presentation. (**I**) Overlap between ATAC-Seq peaks (*P < 0.05) and KDM6B ChIP-Seq binding sites. **P < 0.01 by 2-tailed *t* test. Three replicates were combined for analysis.

To understand the underlying mechanisms, we performed assay for transposase-accessible chromatin sequencing (ATAC-Seq) on WT and *Kdm6b*-deficient Tregs with or without TCR agonist stimulation (Figure 5, A–C, and Supplemental Table 5). In WT Tregs, TCR stimulation induced robust increases in chromatin accessibility at multiple loci that are enriched for transcription factor motifs downstream of TCR signaling, such as AP-1 proteins (Figure 5B). In contrast, *Kdm6b*-deficient Tregs exhibited impaired TCR-induced chromatin opening (Figure 5A and 5C), indicating KDM6B is required for optimal accessibility following activation. Although many regions remained inaccessible, a subset of loci in *Kdm6b*-deficient Tregs showed increased accessibility, especially in the absence of TCR stimulation (Figure 5, A and C), suggesting KDM6B regulates chromatin architecture in both directions, directly and indirectly.

The accessible regions in WT Tregs were enriched for motifs of ETS, JUN/AP-1, and other bZIP transcription factors (Figure 5, B and C), consistent with activation-linked chromatin remodeling downstream of TCR signaling (27, 28, 34, 35). ETS factors cooperate with FOXP3, RUNX, and AP-1 to maintain Treg identity and stability and to restrain excessive effector differentiation (27, 28). Remarkably, *Kdm6b*-deficient Tregs exhibited enrichment of CTCF and IRF motifs regardless of TCR stimulation. CTCF motif is typically associated with constitutively accessible chromatin and higher-order genome organization (36, 37). These results suggest an important role of KDM6B in mediating the impact of ETS, JUN/AP-1, and other transcription factors on chromatin architecture, which is more pronounced after TCR stimulation.

In line with these findings, a proportion of transcripts are associated with chromatin accessibility in the presence or absence of TCR stimulation (Figure 5, D–G). These include list of Treg functional genes, such as *Il1rl1*, *Ikzf2*, *Irf4*, *Jun*, *Ccr5*, *Ccr6*, and *Areg* (Figure 5, D–H). Genes involved in effector T cell programs, such as *Jak1*, *Il5*, *Il31*, and *Ifng* (Supplemental Figure 3), were upregulated along with increased accessibility in *Kdm6b^ΔTreg^* Tregs. However, there was little overlap between TCR-induced increases in chromatin accessibility in *Kdm6b*-deficient versus WT Tregs and KDM6B binding sites (Supplemental Figure 3), suggesting an indirect effect of *Kdm6b* deficiency in promoting chromatin accessibility and transcription. Additionally, there was less than 20% overlap between TCR-induced increases in chromatin accessibility in WT versus *Kdm6b*-deficient Tregs and KDM6B binding sites (Figure 5I), further suggesting direct and indirect effects of KDM6B in promoting chromatin accessibility and transcription.

Overall, ATAC-Seq analysis revealed an important role for Kdm6b in chromatin remodeling and transcriptional regulation in Tregs, particularly after TCR stimulation.

### KDM6B is required for the full function of lung Tregs after injury.

**Figure 6:**
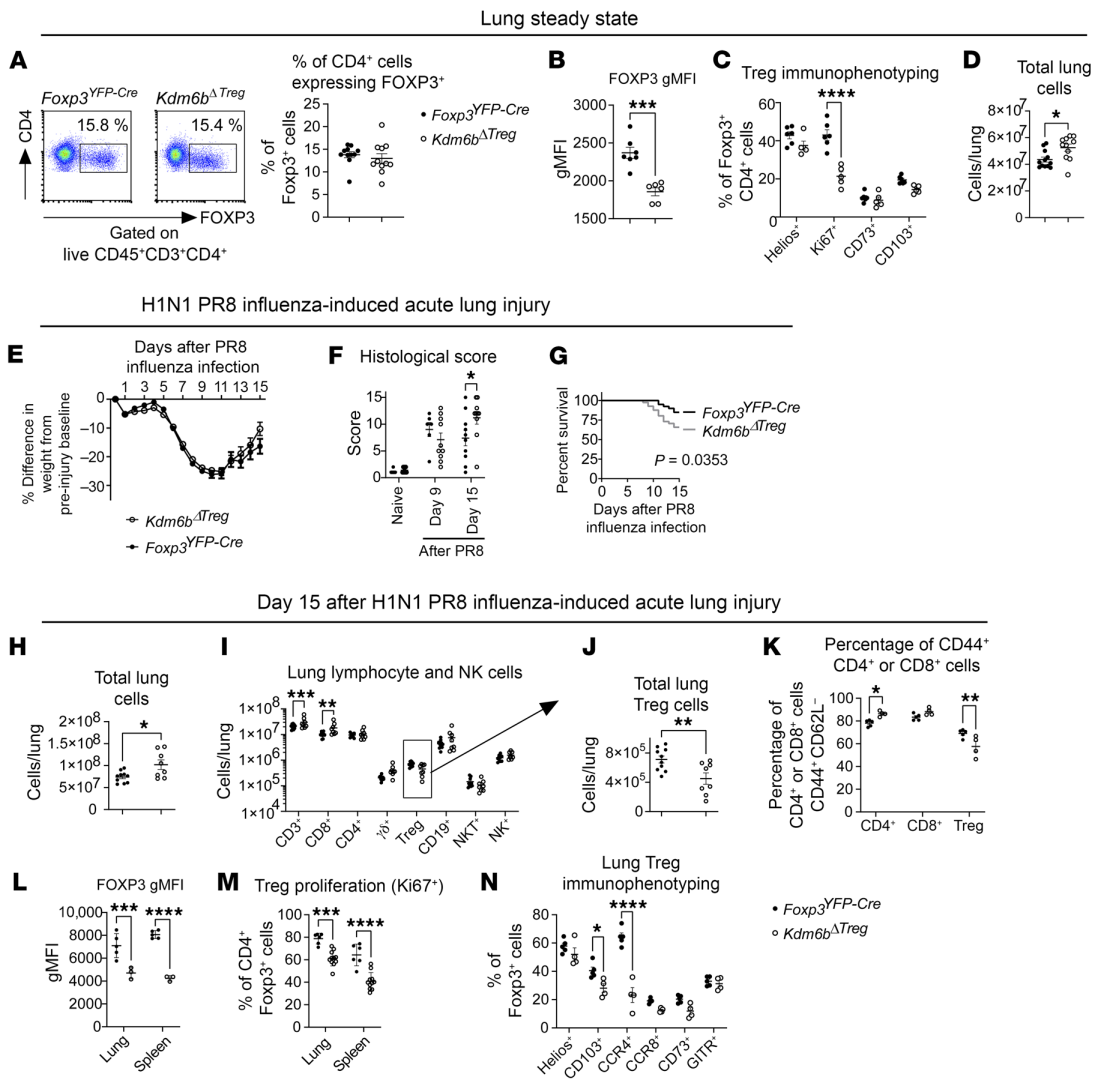
KDM6B is required for full-lung Treg function after injury. Male and female *Foxp3^YFP-Cre^* and *Kdm6b^ΔTreg^* mice were analyzed at steady state or after PR8 influenza challenge on day 0 (2 μL/g body weight). (**A**–**C**) Lung Tregs from single-cell suspensions at steady state were analyzed for frequency of FOXP3^+^ Tregs among CD4^+^ lymphocytes (*n =* 11 mice/strain, ≥2 experiments) (**A**), FOXP3 expression (gMFI) (**B**), and Treg immunophenotype (*n =* 5–6 mice/strain, ≥2 experiments) (**C**). (**D**) Total lung cell counts from enzymatic digests at steady state (*n =* 11–12 mice/strain, ≥2 experiments). (**E**) Percent body weight change after PR8 influenza infection relative to baseline (*n =* 34–35 mice/strain/time point, pooled from ≥3 experiments). (**F**) Histopathology scores from H&E-stained lung sections in *Foxp3^YFP-Cre^* (closed circles) or *Kdm6b^ΔTreg^* mice (open circles) at steady state, day 9, or day 15 after PR8 influenza infection (*n =* 6–10 mice/strain/time point, ≥2 experiments). (**G**) Survival after PR8 influenza challenge (*n =* 40–41 mice/strain, ≥2 experiments). (**H**–**N**) Analyses were performed on day 15 after PR8 influenza infection. (**H**) Total lung cell counts. (**I**) Numbers of lymphocyte and NK subsets assessed using established gating strategies (38, 53) (*n =* 8–10 mice/strain, ≥2 experiments). (**J**) Lung Tregs quantified separately from (**I**). (**K**) Frequency of CD44^+^CD62L^–^ cells among CD4^+^, CD8^+^, or CD4^+^FOXP3^+^ T cells (*n =* 4–5 mice/strain). (**L**–**N**) (**L**) CD4^+^FOXP3^+^ cells from lung or spleen analyzed for FOXP3 expression (gMFI; *n =* 3–5 mice/strain), (**M**) proliferation (*n =* 6–10 mice/strain), and (**N**) lung Treg immunophenotyping (n = 4–5 mice/strain). Data are given as the mean ± SEM. Statistical tests: 2-way ANOVA with Holm-Šidák correction (**C**, **F**, **I**, and **K**–**N**), unpaired 2-tailed *t* test (**A**, **B**, **D**, **H**, and **J**), and log-rank test (**G**). *P < 0.05, **P < 0.01, ***P < 0.001, and ****P < 0.0001.

Given that *Kdm6b* is differentially regulated in lung Tregs during resolution of lung injury compared with steady-state lung Tregs (14), we examined *Kdm6b*-deficient Tregs in lung injury. Steady-state comparison between *Kdm6b^ΔTreg^* Tregs and WT Tregs evaluated in single-cell lung suspensions demonstrated a similar frequency of FOXP3^+^ Tregs (as a percentage of CD4^+^ lymphocytes) and a decrease in Foxp3 expression measured by MFI (Figure 6, A and B). Further immunophenotypic analysis of lung Tregs demonstrated fewer Tregs expressing Ki-67 in CD4^+^FOXP3^+^ Tregs in *Kdm6b^ΔTreg^* mice (Figure 6C). Enumerating total lung cell numbers from enzymatically digested lung single-cell suspensions showed an increased total cell number in *Kdm6b^ΔTreg^* mice (Figure 6D). As shown in Figure 2, E and F, increased numbers of CD3^+^ T cells and an increased proportion of activated CD44^hi^CD62L^lo^ CD4^+^ and CD44^hi^CD62L^lo^ CD8^+^ cells in the lung at steady state were found.

To examine the effects of *Kdm6b* deletion during acute inflammation, we used a mouse-adapted H1N1 influenza model of ALI (38). Weight loss and recovery after inoculation did not differ between strains (Figure 6E). Lung inflammation determined by histological scoring at steady state, during peak injury (day 9 after PR8 influenza infection), and at a later point of resolving injury (day 15 after PR8 influenza infection) demonstrated an appreciable difference between *Foxp3^YFP-Cre^* and *Kdm6b^ΔTreg^* mice at day 15 after PR8 influenza infection (Figure 6F, Supplemental Figure 4, and Supplemental Table 6). *Kdm6b^ΔTreg^* mice did have an increased mortality rate (Figure 6G) and higher total lung cell counts at day 15 after PR8 influenza infection compared with WT *Foxp3^YFP-Cre^* mice (Figure 6H). No difference was detected in viral level or clearance as measured by flow cytometric PR8 nucleoprotein positivity in type II alveolar epithelial cells (Supplemental Figure 5A). There were fewer CD4^+^FOXP3^+^ Tregs at several time points of injury in both BAL and single-cell lung suspensions at 10 days after inoculation in *Kdm6b^ΔTreg^* compared with *Foxp3^YFP-Cre^* mice (Supplemental Figure 5, B and C).

Immunophenotyping lung lymphocytes demonstrated increased total CD3^+^ and CD8^+^ T cells at day 15 after inoculation (Figure 6I). An unpaired *t* test indicated fewer total lung Tregs in *Kdm6b^ΔTreg^* mice at day 15 after PR8 influenza–induced ALI (Figure 6J). Additionally, the proportion of activated CD44^hi^CD62L^lo^ effector CD4^+^ T cells was greater in *Kdm6b^ΔTreg^* mice, and the proportion of activated CD44^hi^CD62L^lo^ Tregs was less (Figure 6K).

Expression of FOXP3 was lower in *Kdm6b^ΔTreg^* mice in both lung and spleen Tregs at day 15 after PR8 influenza–induced ALI, along with a lower proportion of proliferating Tregs identified by Ki-67 expression (Figure 6, L and M). Further lung Treg immunophenotyping demonstrated decreased proportions of CD103^+^ and CCR4^+^ Tregs in *Kdm6b^ΔTreg^* mice (Figure 6N).

Thus, KDM6B promotes lung Treg activation and function during infection. The typical early Treg expansion seen in resolving ALI (38) is absent in *Kdm6b^ΔTreg^* mice in the PR8 influenza model, likely driving increased lung inflammation at day 15 after infection.

### Loss of Kdm6b in Tregs exacerbates dextran sulfate sodium colitis.

To determine whether KDM6B is required for Treg function during acute inflammation in other tissues, we evaluated its role in a dextran sulfate sodium–induced (DSS-induced) colitis model. *Kdm6b^ΔTreg^* mice had significantly shorter colon lengths at study day 11 as compared with *Foxp3^YFP-Cre^* controls, suggesting greater intestinal inflammation (Supplemental Figure 6). Immunophenotyping of mesenteric lymph nodes revealed a reduction in CD44^hi^CD62L^lo^ effector Tregs in *Kdm6b^ΔTreg^* mice, accompanied by decreased frequencies of ICOS^+^, CTLA4^+^, CD103^+^, and Ki67^+^ proliferating Tregs in *Kdm6b^ΔTreg^* mice, whereas expression of GATA3^+^, CD73^+^, and CXCR3^+^ remained comparable between genotypes. These data show KDM6B is necessary for effector Treg function in controlling intestinal inflammation during DSS colitis, highlighting its role in Treg activity across inflammatory contexts.

### KDM6B controls activation-induced H3K27me3 and Treg function in the lung.

**Figure 7:**
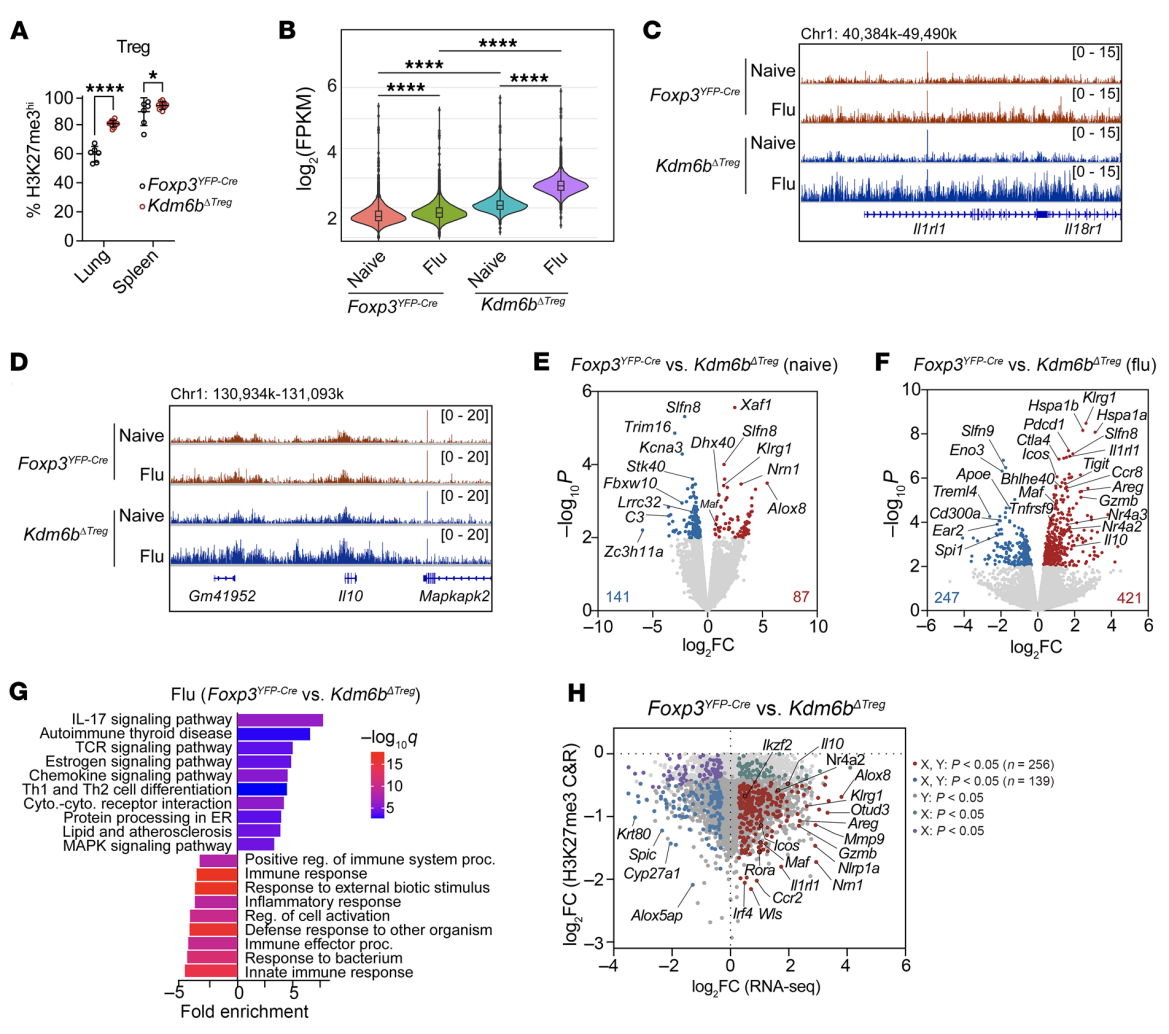
KDM6B controls activation-induced global H3K27me3 level and Treg function in lung Tregs. Tregs from lung and spleen single cell suspensions from male and female *Foxp3^YFP-Cre^* and *Kdm6b^ΔTreg^* mice, 8–12 weeks of age, were sorted at either steady state or after being challenged with intratracheal PR8 influenza as previously described (38) at day 15 after PR8 influenza infection and used for either H3K27me3 CUT&RUN-Seq or RNA-Seq. (**A**) Frequency of H3K27me3^hi^ Tregs from the spleen or lung of *Foxp3^YFP-Cre^* and *Kdm6b^ΔTreg^* mice (*n =* 6–11 mice per strain; a combination of 2 separate experiments). (**B**) Log_2_ FPKM of H3K27me3 signal across the entire genome of lung Tregs from naive and influenza virus–infected *Foxp3^YFP-Cre^* and *Kdm6b^ΔTreg^* mice. Triplicates per condition. (**C** and **D**) H3K27me3 modification tracks in lung Tregs from naive and influenza virus–infected *Foxp3^YFP-Cre^* and *Kdm6b^ΔTreg^* mice. Data from triplicates per condition were combined for analysis and presentation. (**E** and **F**) Volcano plots showing differential gene expression between pairwise conditions of sorted lung Tregs from either *Foxp3^YFP-Cre^* or *Foxp3^ΔKdmb6^* at either (**E**) steady state (naive) or (**F**) day 15 after influenza (Flu) are shown. DEGs significantly (*P* < 0.01) upregulated (red) or downregulated (blue) are shown. Data from triplicates per condition were combined for analysis. (**G**) Gene-set enrichment analysis identified several Gene Ontology biological pathways in sorted *Foxp3^YFP-Cre^* or *Foxp3^ΔKdmb6^* lung Tregs at day 15 after influenza infection. reg, regulation; proc, process. (**H**) Cross-comparison of RNA-Seq and H3K27me3 CUT&RUN results from lung Tregs. Red dots highlight differentially expressed transcripts showing increased expression and decreased H3K27me3 marks in Tregs from *Foxp3^YFP-Cre^* mice compared with *Foxp3^ΔKdmb6^* mice.

Global H3K27me3 determined from flow cytometric analysis of lung and splenic Tregs with deletion of *Kdm6b* (*Kdm6b^ΔTreg^*) showed a dramatic increase compared with WT Tregs (*Foxp3^YFP-Cre^* mice) (Figure 7A). To determine if the changes seen in gene expression in *Kdm6b*-deficient Tregs and the Treg response to inflammation in *Kdm6b^ΔTreg^* mice result from lack of KDM6B-mediated H3K27me3 demethylation, we used H3K27me3 CUT&RUN to identify differences in H3K27me3 marks across the genome in the presence or absence of KDM6B in Tregs. WT and *Kdm6b*-deficient Tregs were sorted from lungs at a steady state or 15 days after PR8 influenza infection. The log_2_ fragments per kilobase per million mapped reads (FPKM) of H3K27me3 signal across the entire genome was quantified in lung Tregs from naive and influenza virus–infected *Kdm6b^ΔTreg^* mice. These data demonstrated a significant increase of H3K27me3 in *Kdm6b^ΔTreg^* compared with WT Tregs (*Foxp3^YFP-Cre^* mice) (Figure 7B and Supplemental Table 7). From this dataset, visualized H3K27me3 modifications for several genes associated with Treg activation and function (*Il1rl1*, *Il18r1*, and *Il10*) are shown (Figure 7, C and D), confirming the role of KDM6B in restricting H3K27me3 modification in Tregs upon immune activation.

Additionally, lung Tregs from WT or *Kdm6b^ΔTreg^* mice from the same experiments used for the CUT&RUN method were examined for differential gene expression by RNA-Seq. Volcano plots (Figure 7, E and F, and Supplemental Table 8) show differential gene expression between pairwise conditions of sorted Tregs from *Foxp3^YFP-Cre^* or *Kdm6b^ΔTreg^* mice at either steady-state (naive) or at day 15 after influenza infection. At steady state (naive), 141 genes exhibited decreased expression, whereas 87 genes showed increased expression (*P* < 0.01) in Tregs from *Foxp3^YFP-Cre^* mice compared with *Kdm6b^ΔTreg^* mice (Figure 7E). On day 15 after influenza infection, expression of 421 genes was increased, including tissue-repair function-related genes *Areg*, *Ctla4*, *Il10*, and *Il1rl1*, and 247 genes exhibited decreased expression in Tregs from WT *Foxp3^YFP-Cre^* mice compared with *Kdm6b^ΔTreg^* mice (Figure 7F). Gene-set enrichment analysis of the significantly altered genes at day 15 after influenza infection identified Gene Ontology biological pathways that were significantly enriched in *Kdm6b^ΔTreg^* lung Tregs at day 15 after influenza infection, including the IL-17 signaling pathway, Th1 and Th2 cell differentiation, and chemokine signaling pathway (Figure 7G).

### Integration of transcriptional and epigenetic profiling reveals KDM6B-dependent repression of key effector Treg programs in the lung.

To link transcriptional changes with epigenetic regulation in *Kdm6b*-deficient Tregs, we integrated the RNA-Seq and H3K27me3 CUT&RUN datasets generated from sorted lung Tregs of *Foxp3^YFP-Cre^* and *Kdm6b^ΔTreg^* mice from day 15 after influenza infection. Differential gene expression (RNA-Seq) was plotted against changes in H3K27me3 at corresponding loci (CUT&RUN), enabling identification of genes exhibiting coordinated transcriptional repression and gain of the repressive histone mark (Figure 7H and Supplemental Table 9).

This analysis revealed a subset of 256 genes (highlighted in red in Figure 7H) that displayed significantly increased expression accompanied by decreased H3K27me3 deposition in WT Tregs (*Foxp3^YFP-Cre^* mice) compared with *Kdm6b*-deficient Tregs. Notably, this group included *Icos* and *Ikzf2* (*Helios*), genes previously implicated in Treg activation, stability, and suppressive function. Additional genes within this category included regulators of tissue adaptation and immune modulation, such as *Areg*, *Il10*, *Rora*, *Maf*, *Il1rl1*, and *Ccr2*, suggesting a broader impairment in effector and tissue-resident Treg programs in *Kdm6b*-deficient Tregs. These findings indicate that loss of *Kdm6b* leads to locus-specific accumulation of H3K27me3 that is associated with transcriptional silencing of key effector Treg genes in the lung after injury.

The response to influenza infection resulted in vastly different transcriptional expression in *Kdm6b*-deficient compared with WT Tregs (Supplemental Figure 7A and Supplemental Table 8). These data support that KDM6B acts through H3K27me3 demethylation to affect Treg identity and function, particularly during inflammatory conditions such as viral infection.

Genes regulated by FOXP3, shown by sgNC versus sgFoxp3 (28), or aTreg versus activated “wannabe” Tregs (expressing a *Foxp3* reporter but not functional FOXP3 protein) were compared with those regulated by KDM6B in lung WT or *Kdm6b^ΔTreg^* Tregs from influenza virus–infected mice to identify upregulated and downregulated transcripts (Supplemental Figure 7, B–E). Subsets of genes were regulated in the same direction by KDM6B and FOXP3. Although the overall overlap comprised a relatively small proportion of DEGs — likely reflecting the independent roles of FOXP3 and KDM6B as well as secondary downstream effects — the shared targets are likely functionally significant. Notably, many of these commonly regulated genes are established regulators of Treg function, including *Klrg1, Il10, Ctla4*, and *Cx3cr1*, suggesting that even limited overlap may have a disproportionate biological impact.

Overlaying separate CUT&RUN experiments revealed that FOXP3 and KDM6B bind to the *Foxp3* and *Tnf* loci (Supplemental Figure 7F). However, deletion of *Kdm6b* in lung Tregs did not appear to alter H3K27me3 marks at these regions at steady state or after lung injury (Supplemental Figure 7F), suggesting KDM6B may regulate FOXP3 function independently of its demethylase activity for some genes. Conversely, KDM6B demethylase activity and H3K27me3 marks may influence the expression of other genes, such as *Il10* (Supplemental Figure 7F).

These data collectively demonstrate that activation induces KDM6B to restrict H3K27me3 modification in lung Tregs, which is essential for Treg function, particularly during viral infection. Additionally, there may be both demethylase-dependent and demethylase-independent mechanisms through which KDM6B functions in Tregs.

## Discussion

This study demonstrates that KDM6B, an H3K27me3 demethylase, is essential for maintaining Treg activation, proliferation, and immune homeostasis, particularly in tissue Tregs responding to injury. Using a Treg-specific *Kdm6b* knockout model, the loss of KDM6B resulted in increased H3K27me3 levels and impaired demethylation in splenic and lung Tregs. Consequently, *Kdm6b*-deficient mice had dysregulated immune balance, marked by elevated activated CD4^+^ and CD8^+^ T cells in the spleen and lungs, highlighting KDM6B’s critical role in Treg-mediated immune regulation.

Mixed bone-marrow chimera analyses showed that loss of Kdm6b in Tregs reduced activated and proliferating Tregs in the thymus, lymph nodes, and spleen under homeostatic conditions. Overall, thymic Treg frequency was unchanged, but Kdm6b-deficient thymic Tregs had a selective reduction in FOXP3^+^CD25^–^ cells, indicating a subtype-specific role after Treg induction. In the periphery, Kdm6b-deficient Tregs had increased CD25 expression, suggesting compensatory IL-2 signaling and impaired activation. Thus, while total Treg numbers are preserved, KDM6B is required cell-intrinsically for stage-specific peripheral Treg activation and proliferative fitness in vivo.

Interestingly, despite these immune perturbations, *Kdm6b^ΔTreg^* mice appear grossly similar to control mice up to 18 weeks of age, with no overt signs of autoimmunity. This phenotype aligns with a model suggesting that Tregs are typically endowed with a buffer zone that enables them to withstand mild perturbations (39). KDM6B may also play an additive role with other epigenetic programs during Treg activation. Despite the increased frequency of FOXP3^+^ Tregs in the spleens of *Kdm6b^ΔTreg^* mice, expression of FOXP3 (gMFI) is reduced, suggesting an impairment of FOXP3 expression levels in Tregs in *Kdm6b^ΔTreg^* mice, which may be driven by increased effector cytokine expression (22). These cytokines may also promote the proliferation and/or survival of Tregs at specific time points, thereby increasing overall Treg frequencies despite the intrinsic effects of *Kdm6b* deficiency on Treg activation and proliferation.

An important consideration is the seemingly paradoxical increase in Treg numbers despite reduced proliferative capacity in *Kdm6b*-deficient Tregs. Similar discordance between Treg abundance and functional competence has been reported in other Treg-specific conditional knockout models, such as *Nfkb2* deficiency, in which Tregs accumulate despite impaired suppressive function (40). The increased frequency of *Kdm6b*-deficient Tregs despite reduced proliferation likely reflects a compensatory homeostatic response to impaired suppressive function. The mixed-chimera experiments show preserved Treg representation in the total CD4^+^ T cell population despite reduced Ki-67 expression, suggesting that Treg accumulation can coexist with a less proliferative, functionally compromised state, potentially influenced by altered activation, survival, or metabolic programs.

Consistent with these possibilities, across multiple Gene Ontology analyses, *Kdm6b*-deficient Tregs showed enrichment of immune activation and inflammatory signaling pathways, including TCR, IL-17, cytokine receptor, and MAPK signaling, consistent with a heightened activation state; however, these data remain correlative and do not directly assess apoptotic pathways, define metabolic programs, or compare conventional CD4^+^ T cells, precluding mechanistic conclusions regarding survival, activation-induced cell death, or metabolic remodeling, and they underscore the need for future studies to directly interrogate Treg survival and metabolic fitness. In our study, we did not observe a significant reduction in *Foxp3* transcript levels in *Kdm6b*-deficient Tregs. Neither the RNA-Seq dataset upon in vitro TCR stimulation nor the RNA-Seq analysis of sorted lung Tregs demonstrated differential *Foxp3* mRNA expression between WT and KO Tregs under any conditions. In addition, the mixed bone-marrow chimera indicates that the reduction in FOXP3 protein levels observed in *Kdm6b^ΔTreg^* mice is not maintained in a cell-intrinsic manner.

Prior work has shown that KDM6B can regulate *Foxp3* transcription in CD4^+^ T cells and directly bind the *Foxp3* promoter (41). In this study, loss of *Kdm6b* did not alter H3K27me3 levels at the *Foxp3* locus, suggesting that its effects on *Foxp3* expression may be independent of canonical H3K27 demethylation. Moreover, *Kdm6b* deletion in that context occurred early in CD4^+^ T cell differentiation, leading to altered lineage skewing.

In contrast, *Kdm6b* deletion in our model is driven by *Foxp3*-Cre, occurring after *Foxp3* expression is initiated. Together, these findings suggest KDM6B may exert distinct, stage-specific effects on upstream roles in CD4^+^ T cell differentiation and on downstream effects on Treg stability or FOXP3 protein regulation not mediated by sustained transcriptional repression. The role of KDM6B in Treg function was further examined in the context of ALI induced by influenza infection, which was associated with a greater mortality rate and worsened inflammation at day 15 after infection. Notably, *Kdm6b^ΔTreg^* mice did not exhibit the typical increase in Treg numbers during the resolution phase of lung injury; instead, they had a decrease in Treg proliferation. The observed increase in H3K27me3 marks in lung Tregs from *Kdm6b*-deficient mice suggests the inability to demethylate these histone marks may impair the transcriptional programs necessary for Treg proliferation, expansion, and function during inflammation, and the increase may underscore a shift in Treg phenotype in the absence of *Kdm6b*. The transcriptional analysis revealed broad differences in gene expression between *Kdm6b*-deficient and WT Tregs, particularly after in vitro TCR stimulation. The global upregulation of proinflammatory cytokine genes and other immune activation pathways in *Kdm6b*-deficient Tregs underscores the importance of KDM6B in maintaining Treg anti-inflammatory and suppressive functions.

This integrative analysis of differential gene expression (RNA-Seq) plotted against changes in H3K27me3 enrichment at corresponding loci (CUT&RUN) enabled identification of a subset of genes that displayed significantly reduced expression accompanied by increased H3K27me3 deposition in *Kdm6b*-deficient Tregs. Together, these findings establish a direct epigenetic link between KDM6B activity and transcriptional maintenance of key effector Treg programs in the lung, highlighting KDM6B as a critical regulator of tissue-adapted Treg function during lung inflammation.

These data indicate that KDM6B shapes Treg chromatin accessibility through both activation-dependent and architectural mechanisms. In WT Tregs, enrichment of ETS and AP-1 motifs highlights a TCR-responsive chromatin program that supports FOXP3-dependent transcriptional networks essential for Treg stability and function (27, 28).

Our data highlight a nuanced role for KDM6B in Treg chromatin regulation. Although *Kdm6b* deficiency leads to widespread H3K27me3 accumulation, RNA-Seq revealed unexpected upregulation of many genes. ATAC-Seq demonstrated that a subset of loci becomes more accessible in *Kdm6b*-deficient Tregs, likely through indirect or compensatory mechanisms, which may account for the increased gene expression. These findings reveal that KDM6B facilitates TCR-driven chromatin remodeling and gene expression in Tregs. That loss of *Kdm6b* creates a complex landscape in which repressive H3K27me3 histone marks, reduced chromatin openness, and transcriptional repression coexist with localized increases in chromatin accessibility and transcriptional upregulation, ultimately shaping transcriptional outputs in Tregs. Decreased Treg proliferation in *Kdm6b^ΔTreg^* mice was the consistent phenotype seen at homeostasis and after acute inflammation in both the lung and mesenteric lymph nodes. T cells undergo proliferation upon T cell activation with TCR stimulation and costimulatory signals (42), and prior studies have demonstrated that Ki-67 expression is highest in the G2/M phase (43) and is continuously degraded in G0/G1 (44). KDM6B has recently been demonstrated as an epigenetic factor linked to constitutive FOXP3-chromatin binding (28). The increased demethylase activity seen in resolving lung Tregs is likely induced by environmental cues, such as TCR signaling and costimulation, and/or during cell activation. Moreover, the KDM6B protein was identified as a protein induced by TCR signaling and found near FOXP3 by proximity proteomics (28).

Balanced H3K27 modification is essential for optimal Treg function (26). EZH2, a histone methyltransferase, catalyzes the addition of methyl groups to H3K27, which recruits Polycomb complexes, leading to gene silencing (19). In contrast, KDM6B is a histone demethylase that removes methyl groups from H3K27, activating gene expression. KDM6B facilitates Treg differentiation by removing repressive histone marks from genes associated with Treg lineage specification (20, 41). EZH2 promotes the development of Tregs from precursor cells by repressing genes involved in alternative T cell lineages (26). The absence of EZH2 in T cells results in reduced proliferation during infection and suboptimal inducible Treg generation (22). Two studies have demonstrated that the absence of *Ezh2* leads to the accumulation of proapoptotic mediators, affecting the ability of CD4^+^ lymphocytes to proliferate (45, 46). Mice that lack *Ezh2*, specifically in Tregs, develop autoimmunity (26), and EZH2 has been demonstrated to play a role in Treg homeostasis by regulating effector cytokines, which inhibit the expression of FOXP3 (22). Recent work by Peeters et al. (23) demonstrated that increasing H3K27me3 levels with a gain-of-function mutation in EZH2 led to increased effector Treg phenotypes with enhanced suppression, likely through redistribution of H3K27me3 modifications, similar to what is seen in WT Tregs after CD28 costimulation. Moreover, Tregs expressing this gain-of-function EZH2 had increased capacity to migrate to lung tissue under steady-state conditions (23). Work by Wei et al. (47) demonstrated that the *Foxp3* locus does not have significant H3K27me3; therefore, the effects of EZH2 and KDM6B on *Foxp3* expression may be indirect or independent of enzymatic activity. Because deletion of either *Ezh2* or *Kdm6b* leads to similar phenotypes, more studies are needed to compare the 2 head-to-head to understand this complex interplay.

It remains unclear whether KDM6B primarily functions through demethylase activity or through demethylase-independent mechanisms. Overall, balanced H3K27 regulation by EZH2 and KDM6B is critical for Treg development, function, stability, and immune tolerance.

Although KDM6B and FOXP3 appear to function independently, recent evidence shows that FOXP3 binds chromatin via other transcription factors activated by TCR and cytokine signaling (28). We propose that FOXP3 acts as a transcriptional cofactor that integrates extracellular cues and activation state to regulate Treg genes (27). Supporting this model, KDM6B enables TCR-dependent chromatin accessibility and Treg activation, which FOXP3 monitors and coordinates to control Treg gene expression. This supports a model in which FOXP3 defines Treg identity, and KDM6B enables TCR-dependent chromatin remodeling required for FOXP3 function. KDM6B loss disrupts this cooperation, leading to defective activation-linked accessibility, greater reliance on chromatin architecture, and discordance between repressive histone marks and gene expression in *Kdm6b*-deficient Tregs.

In conclusion, this study demonstrates that KDM6B is a critical regulator of Treg function, at least in part through modulating H3K27me3 marks. The loss of *Kdm6b* in Tregs impairs suppressive function, alters immune homeostasis, and diminishes the ability to respond to inflammatory challenges. These findings have implications for understanding the epigenetic regulation of Tregs and suggest that targeting KDM6B or the pathways it regulates may offer strategies to modulate immune responses in diseases characterized by Treg dysfunction.

## Methods

### Sex as a biological variable.

Our study examined male and female animals, and similar findings were reported for both, except where noted otherwise.

### Reagents and resources.

A list of reagents and commercial assays, along with their catalog numbers, is provided in Supplemental Table 10.

### Mice.

*Kdm6b^fl/fl^* mice (31) were crossed with *Foxp3^YFP-Cre^* mice (9) to generate Treg-specific *Kdm6b* knockout mice (*Kdm6b^ΔTreg^*). Mice were maintained under specific pathogen–free conditions at UNC at Chapel Hill. All experiments were approved by the UNC Institutional Animal Care and Use Committee in accordance with NIH guidelines. In experiments, similar numbers of male and female mice from each strain and condition were used, with experiments carried out at time points as described.

### Initiation of inflammation by influenza.

Equal numbers of male and female mice, 8–12 weeks of age, were anesthetized with tribromoethanol before tracheal intubation and administration of influenza, as previously described (38). Influenza A/PR/8/34 H1N1 (PR8) was purchased from Charles River. The viral administration has been dose optimized for eliciting an inflammatory response with modest mortality, facilitating a better study of the resolution phase of ALI (48). The virus was suspended, diluted in PBS, and stored at −80°C at a 2 × 10^8^ egg-infective dose/mL. Pneumonia was induced by intratracheal instillation of the thawed viral suspension diluted in mice received 2 μL/g of thawed viral suspension diluted in PBS to 2.5 × 10^6^ egg-infective dose/mL for weight-based dosing, as previously described (38).

### DSS colitis.

DSS salt was dissolved in autoclaved drinking water to a concentration of 3% wt/vol and provided to female mice for 10 days. Fresh DSS-supplemented water was replaced every 2–3 days, and body weight was monitored daily throughout the treatment period.

### Lung morphology.

At euthanasia, lungs were inflated via the trachea to 25 cm H_2_O with 10% neutral buffered formalin, fixed for 48–72 hours, processed to paraffin, sectioned at 4 μm, and stained with H&E. A masked, board-certified veterinary pathologist performed qualitative and semiquantitative histologic scoring (Supplemental Table 6). Alveolar hyaline membranes, airway epithelial changes, epithelial injury and regeneration, and perivascular and peribronchiolar inflammation were scored on a scale from 0 to 5 (0 = none; 5 = severe). Bronchointerstitial pneumonia and fibrosis were scored by percentage of lung involvement (range: 1–5, where 1 = <10% and 5 = 76%–100%). Scores were compared across study days and groups, including a combined qualitative score.

### Analysis of bronchoalveolar lavage fluid.

At euthanasia, bronchoalveolar lavage (BAL) fluid was collected by tracheal cannulation with a 20-gauge catheter. Both lungs were lavaged twice with 1 mL of calcium-free PBS. BAL was centrifuged at 400*g* for 5 minutes at 4°C; supernatants were then centrifuged at 1,300*g* to remove debris and stored at −80°C. Cell pellets were resuspended in PBS, and viable cells were counted by trypan blue exclusion. BAL flow cytometry was performed as described under *Multicolor flow cytometry for cell analysis*. Total protein in cell-free supernatant was measured using a DC protein assay.

### In vitro suppression assay.

Tregs and CD4^+^ effector splenocytes were cultured with CD3/CD28 beads in RPMI 1640 medium supplemented with 10% FBS, l-glutamine, sodium pyruvate, nonessential amino acids, HEPES, penicillin-streptomycin, human IL-2 (50 U/mL), and β-mercaptoethanol (50 μM), as previously described (38). Tregs were isolated from spleens using magnetic separation, and responder CD4^+^ splenocytes were CFSE-labeled prior to coculture (6). Suppression assays were maintained for 3 days with media refresh and splitting as needed. Cells were then Fc blocked, stained for CD4^+^ and viability, and analyzed by flow cytometry. CFSE dilution in responder cells was used to assess proliferation.

### Mixed bone-marrow chimeric mice experiment.

Bone marrow was obtained and mixed bone-marrow chimeric mice were generated as previously described (49). Briefly, recipient mice (CD45.1; CD45.2) were irradiated (9.5 Gy) 1 day before transferring 10^6^ bone marrow cells from CD45.1 WT and CD45.2 *Kdm6b^ΔTreg^* mice mixed at a 1:1 ratio. After bone marrow transfer, the recipient mice were administered 2 mg/mL neomycin in drinking water for 3 weeks and analyzed 8 weeks later.

### Preparation of lung single-cell suspensions for flow cytometry analysis and cell sorting.

Lungs were digested by intratracheal instillation of 1 mL of 5 mg/mL collagenase I and 0.25 mg/mL DNase I in RPMI medium via a 20-gauge catheter, followed by 0.5 mL of 1% (wt/vol) low-melting agarose, as described previously (6). Lungs were incubated at 37°C for 20 minutes, then minced and triturated through an 18 gauge needle. Cell suspensions were then filtered through a 100 μM filter before RBC lysis and stained, as previously described (16).

### Multicolor flow cytometry for cell analysis.

Single-cell suspensions were prepared in FACS buffer, and total cell counts were determined by hemocytometer. Cells were Fc blocked (anti-CD16/32) and surface stained with fluorochrome-conjugated antibodies prior to fixation and permeabilization using the Foxp3/Transcription Factor Staining Buffer Set (eBioscience), similarly to what has been described elsewhere (6). Where indicated, cells were stimulated for 6 hours with PMA/ionomycin in the presence of brefeldin A for intracellular cytokine detection. Flow cytometry was performed on a Cytoflex cytometer and analyzed using CytExpert software (both Beckman Coulter).

### Multicolor flow cytometric cell sorting.

For Treg sorting, single-cell digests from lungs of *Foxp3^YFP-Cre^* mice or *Kdm6b^ΔTreg^* mice were suspended in a FACS buffer containing 33% Percoll (Sigma). The cell suspension was then centrifuged (930*g* at 4°C for 20 minutes with low acceleration and deceleration), and the cell pellet containing lymphocytes was resuspended in FACS buffer. Cells then underwent Fc-receptor blockade with rat anti-mouse FcgRIII/II receptor (CD16/32) and surface-stained with Alexa 700 conjugated anti-mouse CD4. Cells were gated and sorted for CD4^+^YFP^+^ cells (Tregs) using a FACSAria instrument and FASCDiva software (Becton Dickinson), and analyzed using FlowJo (Tree Star) software. After sorting, Tregs were flash-frozen in liquid nitrogen and later used for either RNA purification or H3K27me3 CUT&RUN.

### RNA isolation.

RNA was isolated from approximately 250,000 sorted Tregs (CD4^+^YFP^+^) from lung, spleen, lymph nodes, or influenza-infected tissue of *Foxp3^YFP-Cre^* or *Kdm6b^ΔTreg^* mice. Snap-frozen cells were lysed in TRI Reagent or TRIzol, and RNA was extracted using Directzol or standard TRIzol protocols (14). RNA was eluted in DNase/RNase–free water, quantified by NanoDrop (Thermo Fisher), quality checked by Agilent Bioanalyzer, and stored at −80°C prior to RNA-Seq. rTregs were defined as CD4^+^YFP^+^CD44^lo^ CD62L^hi^, and the subset was stimulated with anti-CD3/CD28 for 3 hours before lysis.

### CUT&RUN.

H3K27me3 CUT&RUN was performed using the CUT&RUN Assay Kit (Cell Signaling Technology) following the manufacturer’s instructions and prior reports (50). Approximately 250,000 sorted lung or splenic Tregs (CD4^+^YFP^+^) from *Foxp3^YFP-Cre^* or *Kdm6b^ΔTreg^* mice (steady state or after PR8 influenza) were used. Cells were bound to concanavalin A beads, permeabilized with digitonin, and incubated overnight at 4°C with anti-H3K27me3 antibody. Beads were incubated with protein A/G–MNase, activated with CaCl_2_, and reactions stopped with buffer containing bacterial spike in DNA. DNA was purified using the DNA Purification Buffer and Spin Column Kit (Cell Signaling Technology). Libraries were sequenced by Asenat (ChIP-Seq platform), and sequencing reads were mapped to the mouse genome (mm10).

KDM6B CUT&RUN was performed as previously described with minor modifications (28). Briefly, 250,000 cells were permeabilized and incubated overnight at 4°C with anti-KDM6B antibody (Abcam). Following incubation with protein A/G–MNase, digestion was initiated with CaCl_2_ and stopped with STOP buffer. DNA was recovered by phenol-chloroform extraction and ethanol precipitation, and libraries were prepared using the KAPA HyperPrep kit (Roche). Sequencing reads were mapped to the mouse genome (mm10).

### ATAC-Seq.

rTregs (CD4^+^YFP^+^CD44^lo^ CD62L^hi^) were sorted from spleens and lymph nodes of 11- to 14-week-old male *Foxp3^YFP-Cre^* mice or *Kdm6b^ΔTreg^* mice. A set of sorted Tregs was incubated with 1 μg/mL anti-CD3 and anti-CD28 antibodies for 3 hours. After stimulation, 50,000 cells were washed with 1× cold PBS. Then, 300 μL of lysis buffer (10 mM Tris-HCl [pH 7.4], 10 mM NaCl, 3 mM MgCl_2_, 0.1% MP-40, molecular-grade H_2_O) was added to wells and centrifuged at 1,200*g* for 10 minutes in a swinging-bucket centrifuge. After removal of supernatant containing lysis buffer and cell debris, the nuclei pellet was then resuspended in 50 μL of tagmentation reaction mix containing molecular grade H_2_O and 2× Tagment DNA Buffer (Illumina), followed by the addition of 2.5 μL of TDE1 (Illumina), and incubated at 37°C for 0.5 hours. The transposed DNA was cleaned up with a Qiagen MinElute PCR Purification Kit. The library preparation and deep sequencing were performed as described elsewhere (28).

### Data analyses.

CUT&RUN data analyses, ATAC-Seq data analyses, motif extraction, and RNA-Seq data analyses were performed as described previously (28). Functional annotation of genes was performed with ShinyGO 0.80 (http://bioinformatics.sdstate.edu/go80/) (51).

### In vivo CRISPR screening.

A retroviral sgRNA library targeting the epifactor coding genes was constructed with the pSIR-BbsI-Thy1.1 backbone (30). We selected 4 sgRNAs for each gene, and 100 nontargeting sgRNAs were used as NCs. *Il2ra* and *Zap70,* with reported roles in Treg function or TCR signal transduction, were included.

To perform CRISPR screening in vivo, on day 0, natural Tregs were double sorted by FACS from *Rosa^Cas9/+^ Foxp3^gfp^* mice and seeded on 24-well plates at 1 × 10^6^ to approximately 2 × 10^6^ cells/well together with Mouse T-Activator CD3/CD28 beads (Thermo Fisher Scientific) and 1,000 U/mL recombinant human IL-2. On day 3, retrovirus was added for transduction, with an anticipated efficiency of 10%–15%. Transduction was performed as described earlier in Methods in the presence of 6 μg/mL polybrene and 10 mM HEPES by centrifuging at 1,200*g* at 35°C for 90 minutes. After transduction, the culture medium was changed to fresh, complete RPMI 1640 medium containing 1,000 U/mL IL-2. At 18 hours after transduction, on day 4, cells were expanded onto larger plates with a 2-fold larger surface area with fresh medium and Mouse T-Activator CD3/CD28 beads. A small fraction of cells was collected and kept at –80°C as day 4 input. From day 0 to day 7, fresh medium with 1,000 U/mL recombinant human IL-2 was replenished every 2 days. On day 7, GFP^+^ Tregs were sorted, and a small aliquot was kept as day 7 input. The remaining GFP^+^ Tregs were cotransferred with CD4 T cells, isolated from CD45.1 mice with the EasySep Mouse CD4 naive T Cell Isolation Kit (STEMCELL), into *Rag1^–/–^* mice. Each *Rag1^–/–^* mouse received 0.5 × 10^6^ Tregs and 2 × 10^6^ CD45.1^+^ CD4 T cells.

Two weeks later, lymphocytes were recovered from the spleen, skin-draining lymph nodes, mesenchymal lymph nodes, liver, lung, and small and large intestines according to protocols described previously (49). Briefly, the liver and lung were perfused with cold PBS and digested with collagenase type 4 (1 mg/mL) at 37°C for 15–45 minutes. For intraepithelial lymphocyte and lamina propria lymphocyte isolation, the small and large intestines were chopped into 2–3 cm fragments, washed twice with cold PBS, and incubated in PBS containing 1 mM EDTA and 10% FBS at 37°C with shaking for 15 minutes to release intraepithelial lymphocytes. The remaining tissues were digested with 1 mg/mL collagenase in RPMI 1640 medium containing 10% FBS at 37°C for 30 minutes. Cell suspensions were filtered through a 100 mm filter, and lymphocytes were separated by differential Percoll centrifugation. Purified lymphocytes were stained, and CD45.1^–^CD45.2^+^TCRβ^+^GFP^+^ Tregs were sorted by FACS to analyze sgRNA representation.

### sgRNA recovery.

To recover sgRNA sequences, genomic DNA was isolated from sorted T cells by proteinase K digestion followed by phenol-chloroform extraction and isopropanol precipitation. sgRNA cassettes were amplified using a 2-step PCR. The first PCR (PCR1) amplified the sgRNA region from the retroviral backbone and incorporated adaptor sequences, and the second PCR (PCR2) added Illumina i5/i7 barcodes. PCRs were performed as previously described (52) using Q5 Hot Start HiFi polymerase (New England Biolabs). PCR1 products were purified, and aliquots were used as templates for PCR2. Final amplicons (261 bp) were gel purified and sequenced on an Illumina MiSeq (single-end, 100 cycles), yielding more than 1 × 10^6^ reads per sample.

### sgRNA library design and data analysis.

The sgRNA library was designed using the Broad GPP sgRNA Designer rule set (51). Briefly, 4 sgRNAs were designed per target gene along with 100 NCs. Next, for library amplification and cloning, the adapter sequences 5′ TATCTTGTGGAAAGGACGAAACACCG 3′ and 5′ GTTTTAGAGCTAGAAATAGCAAGTTAAAAT 3′ were appended to the 5′ and 3′ sgRNA sequence, respectively. The full sgRNA library was ordered from TWIST Bioscience. To determine the differential representation of sgRNAs after in vivo selection, the FASTQ data were demultiplexed using MiSeq Reporter software (Illumina).

Paired-end reads were trimmed, filtered, and mapped to the sgRNA library using CLC Genomics Workbench, version 12 (Qiagen). sgRNA counts were normalized to total reads from 100 nontargeting sgRNAs per sample. To minimize clonal effects, counts from 4 sgRNAs targeting the same gene were combined, and enrichment in recovered versus input cells was assessed by paired Student’s *t* test and log_2_ fold change. As a control, 100 nontargeting sgRNAs were randomly grouped into 25 sets of 4, and *P* values and log_2_ fold changes were calculated correspondingly.

### Statistics.

Statistical analyses were performed using GraphPad Prism version 10, with a P value of less than 0.05 considered significant. Normality was assessed by the Shapiro–Wilk test. Data are shown as mean ± SEM unless otherwise noted, and statistical tests are specified in the corresponding figure legends. For comparisons between 2 groups, unpaired 2-tailed Student’s *t* tests were applied where appropriate. For experiments involving multiple groups or variables, 1-way or 2-way ANOVA was used, followed by appropriate post hoc multiple-comparison tests (Holm-Šídák). For survival Log-rank test was used. The choice of statistical test was based on the experimental design and data characteristics, and all tests used were appropriate for the analyses performed.

### Study Approval.

The UNC Animal Care and Use Committee approved procedures and protocols.

### Data availability.

Further information and requests for resources and reagents should be directed to and will be fulfilled by the lead contact, Jason R. Mock (jason_mock@med.unc.edu). Values for all data points in graphs are reported in the Supporting Data Values file. RNA-Seq and H3K27me3 and KDM6B CUT&RUN sequencing data have been deposited in the Gene Expression Omnibus under accession code GSE291449. No original code is reported.

## Author contributions

MH, CMD, KBS, AT, YF, and JRM conceptualized the study. Methodology was developed by MH, KBS, YF, and JRM. The formal analysis was conducted by RSS, MH, YF, and JRM. The investigation was carried out by MH, BX, PGB, MJM, XZ, WQ, BLB, MKT, MPZ, BCM, YDW, JAS, SMPM, YF, and JRM. Resources were provided by MH, MPZ, GM, BCM, JJM, KBS, YF, and JRM. Writing of the original draft was completed by CMD and JRM. Writing, review, and editing were performed by MH, PGB, MJM, RSS, MZ, GM, BCM, JJM, JKW, CMD, AT, KBS, YF, and JRM. Funding acquisition and supervision were carried out by YF and JRM.

## Conflict of interest

The authors have declared that no conflicts of interest exist.

## Funding support

This work is the result of NIH funding, in whole or in part, and is subject to the NIH Public Access Policy. Through acceptance of this federal funding, the NIH has been given a right to make the work publicly available in PubMed Central.

- NIH (grants R01HL152077 and R01HL173765 to JRM); T32GM133364 to MPZ, K08CA248960 to BCM; R21AI163942 and R01AI153138 to YF; P30CA021765 to St. Jude Children’s Research Hospital; and P30CA016086 to the UNC Lineberger Comprehensive Cancer Center. Histology services were supported by the UNC–Chapel Hill Pathology Services Core in part through NCI grant P30CA016086.
- The Burroughs Wellcome Fund Career Award provided additional support for BCM.
- The American Lebanese Syrian Associated Charities (St. Jude).

## Supplementary Material

Supplemental data

Supplemental table 1

Supplemental table 2

Supplemental table 3

Supplemental table 4

Supplemental table 5

Supplemental table 6

Supplemental table 7

Supplemental table 8

Supplemental table 9

Supporting data values

## Version 1 04/23/2026

In-Press Preview

## Version 2 07/01/2026

Electronic publication
