# Supplementary material for: T cell receptor signaling induces expression of lysine demethylase KDM6B to maintain Treg homeostasis
Source: J Clin Invest. 2026 Apr 23;136(13):e196022. doi: 10.1172/JCI196022 (PMC13318106; doi:10.1172/JCI196022)
Supplement: Supplemental data [file jci-136-196022-s064.pdf]

## SUPPLEMENTAL MATERIAL

### Supplemental Figures

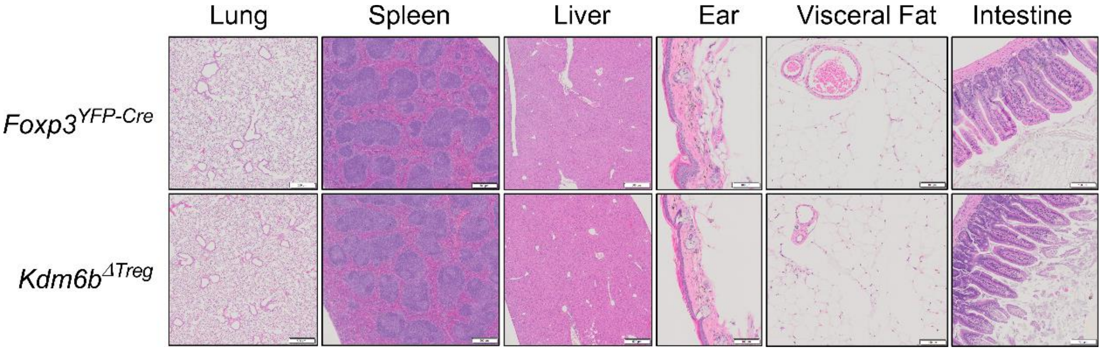


**Supplemental Figure 1. Histological comparison of select organs from *Foxp3*$^{YFP\text{-}Cre}$ or *Kdm6b*$^{\Delta Treg}$ mice.** Histological H&E section of organs from *Foxp3*$^{YFP\text{-}Cre}$ or *Kdm6b*$^{\Delta Treg}$ mice, aged 8-12 weeks. The white bar in the lung, spleen, and liver represents 500 μM, and the white bar in the ear, visceral fat, and intestine represents 100 μM. Sections from organs represent sections from *Foxp3*$^{YFP\text{-}Cre}$ or *Kdm6b*$^{\Delta Treg}$ mice (images representative of n = 3 – 4 mice per strain).

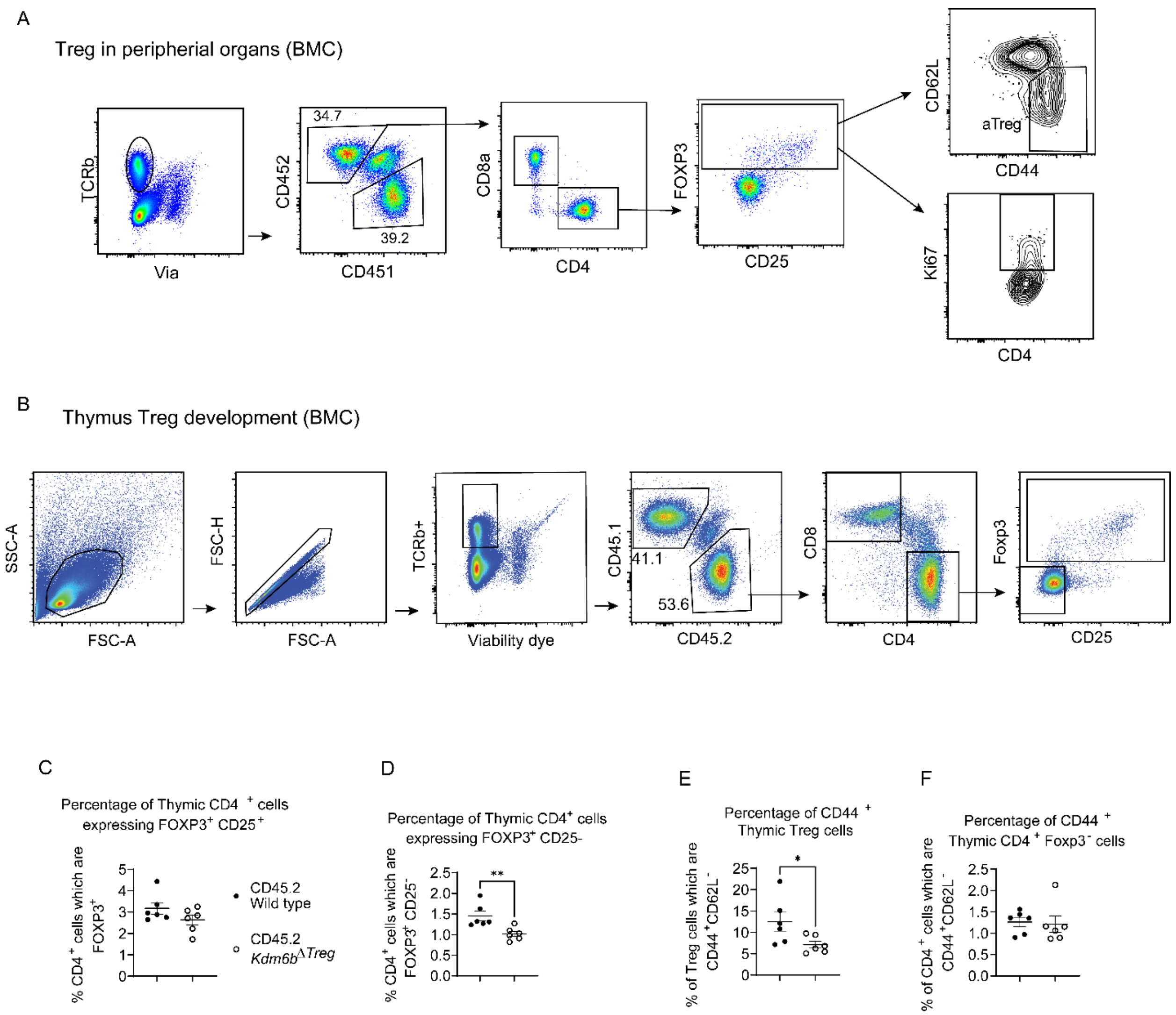


**Supplemental Figure 2. Kdm6b regulates early thymic Treg activation status without altering overall Treg abundance.**

(**A**) Representative flow cytometry gating strategy used to identify Tregs and activated/proliferating subsets in peripheral organs from mixed bone marrow chimeras (BMC). Immunophenotyping of CD4⁺FOXP3⁺ lymphocytes from splenocytes, lymph nodes (LN), or lung single-cell suspensions from a mixed bone marrow chimeric experiment after 8 weeks of engraftment comparing CD45.2⁺ wild-type or CD45.2⁺ *Kdm6b*$^{\Delta Treg}$ Tregs (n = 6 mice per condition).

(**B**) Gating strategy for analysis of thymic Treg development in BMC. (**C–D**) Quantification of the percentage of thymic $CD4^+$ T cells expressing $Foxp3^+$ $CD25^+$ (**C**) or $FOXP3^+$ $CD25^-$ (**D**) populations derived from WT ($CD45.2^+$) or *Kdm6b*$^{\Delta Treg}$ ($CD45.2^+$) donors in BMCs.

(**E**) Percentage of activated thymic Tregs ($CD44^+$ $CD62L^-$) among total $FOXP3^+$ Tregs. (**F**) Percentage of $CD44^+$ cells among thymic $CD4^+$ $FOXP3^-$ T cells. Data presented as mean ± SEM with p values derived from unpaired t-tests, * $p < 0.05$, ** $p < 0.01$

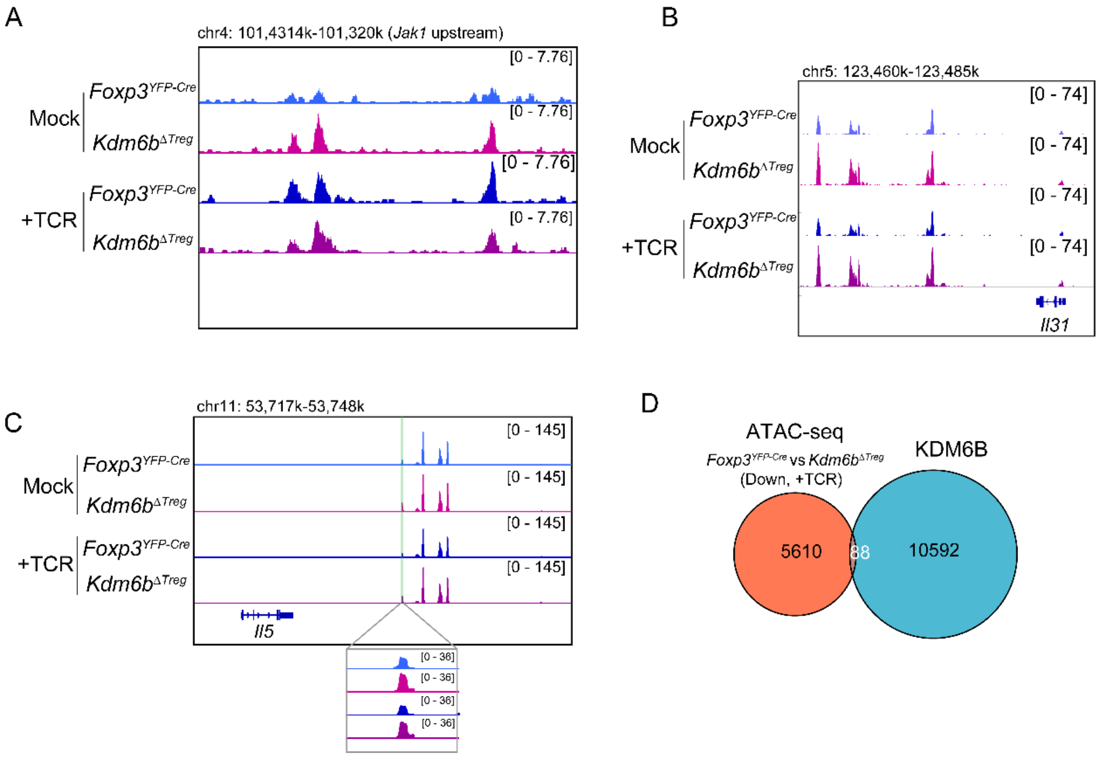


**Supplemental Figure 3. Evaluation of chromatin accessibility at loci related to effector T cells.**

TCR- vs mock-treated WT *Foxp3$^{YFP\text{-}Cre}$* and *Kdm6b$^{\Delta Treg}$* Tregs were subjected to ATAC-seq. Three replicates were combined for analysis. Normalized sequencing reads at the *Jak1* (**A**), *Il31* (**B**), and *Il5* (**C**) loci are shown. (**D**) Overlap between ATAC-seq peaks and Kdm6b ChIP-seq binding sites. Three replicates were combined for analysis.

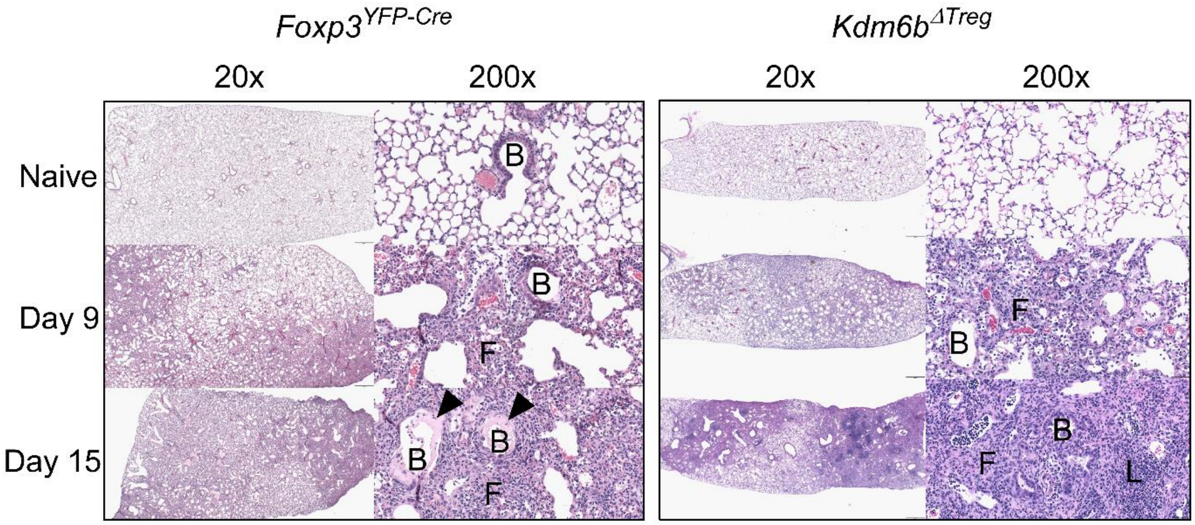


**Supplemental Figure 4. Histological comparison of lung from *Foxp3*$^{YFP\text{-}Cre}$ or *Kdm6b*$^{\Delta Treg}$ mice.** Histological H&E section of a lung from *Foxp3*$^{YFP\text{-}Cre}$ or *Kdm6b*$^{\Delta Treg}$ mice, aged 8-12 weeks at either naïve, day 9 or day 15 post PR8 infection. The black bar at 20x represents 500 μM and 50 μM at 200x. Sections are representative of lung findings from *Foxp3*$^{YFP\text{-}Cre}$ or *Kdm6b*$^{\Delta Treg}$ mice at each time point (at least 5 animals per group). *Foxp3*$^{YFP\text{-}Cre}$ or *Kdm6b*$^{\Delta Treg}$ mice administered influenza virus have a similar area of lung with bronchointerstitial pneumonia at Day 9 post infection, although the *Kdm6b*$^{\Delta Treg}$ mice did equivocally have more widespread inflammation than *Foxp3*$^{YFP\text{-}Cre}$ mice. Inflammation is comprised of neutrophils, macrophages, and lymphocytes with focally extensive areas of alveolar wall fibrosis, reorganizing fibrin, and bronchiolar epithelial cell degeneration, regeneration, and necrosis with bronchiolitis obliterans. At Day 15 post infection, the *Foxp3*$^{YFP\text{-}Cre}$ mice had less lung area with bronchointerstitial inflammation than the *Kdm6b*$^{\Delta Treg}$ mice. The nature of the inflammation was similar to Day 9, but the fibrosis and bronchiolar injury was more extensive. At Day 15, respiratory epithelial cell proliferation was present at a higher incidence and severity in the *Kdm6b*$^{\Delta Treg}$ group compared to the *Foxp3*$^{YFP\text{-}Cre}$ group. Additionally, lymphocytic aggregates were also evident at a higher incidence and severity in the *Kdm6b*$^{\Delta Treg}$ group compared to the *Foxp3*$^{YFP\text{-}Cre}$ group. Hyalinized rings (arrowhead; putatively denuded

bronchioles) were evident more frequently in the *Foxp3*$^{YFP\text{-}Cre}$ group than the *Kdm6b*$^{\Delta Treg}$ group.

B=bronchiole; F=fibrosis; L=lymphocytic aggregates

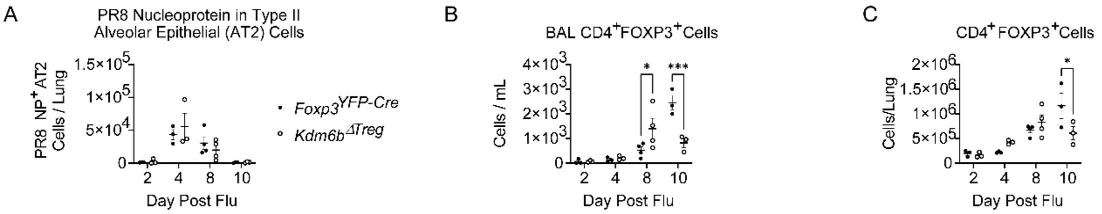


**Supplemental Figure 5. Kinetics of PR8 viral infection and numbers of Tregs in the bronchoalveolar lavage and lung compartments.**

Male and female *Foxp3*$^{YFP\text{-}Cre}$ and *Kdm6b*$^{\Delta Treg}$ mice 8 – 12 weeks of age were examined at either steady state or challenged with intratracheal Influenza A/PR/8/34 H1N1 (PR8) administered at day 0. Mice received 2 μL/g body weight for weight-based dosing as previously described (38). (**A**) Multicolor flow cytometry, as previously described, was used to determine the number of type II alveolar epithelial cells containing PR8 nucleoprotein antigen at several time points post PR8 infection in *Foxp3*$^{YFP\text{-}Cre}$ and *Kdm6b*$^{\Delta Treg}$ mice. (**B-C**) Tregs were enumerated from either bronchoalveolar lavage or lung single-cell suspensions at several time points post PR8 infection in *Foxp3*$^{YFP\text{-}Cre}$ and *Kdm6b*$^{\Delta Treg}$ mice (n = 3-4 mice per strain per time point). Data presented as mean ± SEM with p values derived from two-way ANOVA with Holm-Sidak multiple comparisons. * $p < 0.05$, *** $p < 0.001$.

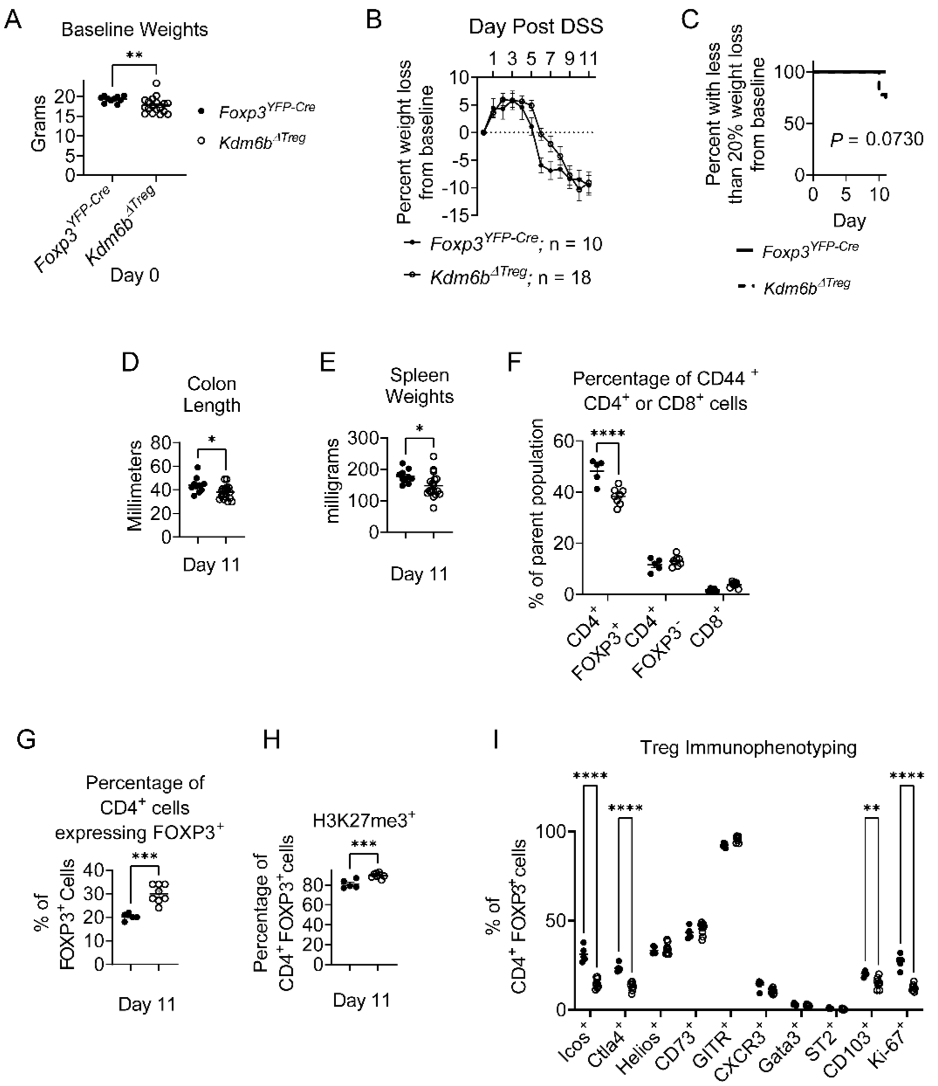


**Supplemental Figure 6. Loss of *Kdm6b* in FOXP3+ Tregs exacerbates DSS colitis and alters Treg phenotype.**

(**A**) Baseline body weights of female *Foxp3$^{YFP\text{-}Cre}$* and *Kdm6b$^{\Delta Treg}$* mice 8 – 12 weeks of age were examined at either steady state then challenged with 3% w/v of dextran sulfate sodium (DSS) in drinking water administered at day 0 as similar to previously described (54) (n = 10-18 mice per strain; combining 2 separate experiments).

(**B**) Percent weight loss from baseline over the DSS course in *Foxp3$^{YFP\text{-}Cre}$* (closed circles) and *Kdm6b$^{\Delta Treg}$* (open circles) mice.

(**C**) Proportion of mice maintaining ≥80% of baseline body weight over time following DSS initiation.

(**D**) Colon length and (**E**) spleen weights were measured at day 11.

(**F-I**) Immunophenotyping of mesenteric lymph nodes at day 11 (n = 5-8 per strain). (**F**) Frequencies of $CD44^+$ effector cells among $CD4^+$ and $CD8^+$ T cells. (**G**) Percentages of $CD4^+$ T cells expressing FOXP3 and (**H**) frequencies of $H3K27me3^+$ cells among $FOXP3^+$ $CD4^+$ T cells (**I**) Expression of canonical activation and lineage-defining markers (ICOS, CTLA4, Helios, CD103, GITR, CXCR3, GATA3, RORγt, CD103, Ki-67) on $FOXP3^+$ $CD4^+$ T cells assessed by flow cytometry. Data presented as mean ± SEM with p values derived from two-way ANOVA with Holm-Sidak multiple comparisons (**B**, **F**, **I**), unpaired t-test (**A**, **D**, **E**, **G**, **H**) and Log-Rank (**C**). * $p < 0.05$, ** $p < 0.01$, *** $p < 0.001$, **** $p < 0.0001$.

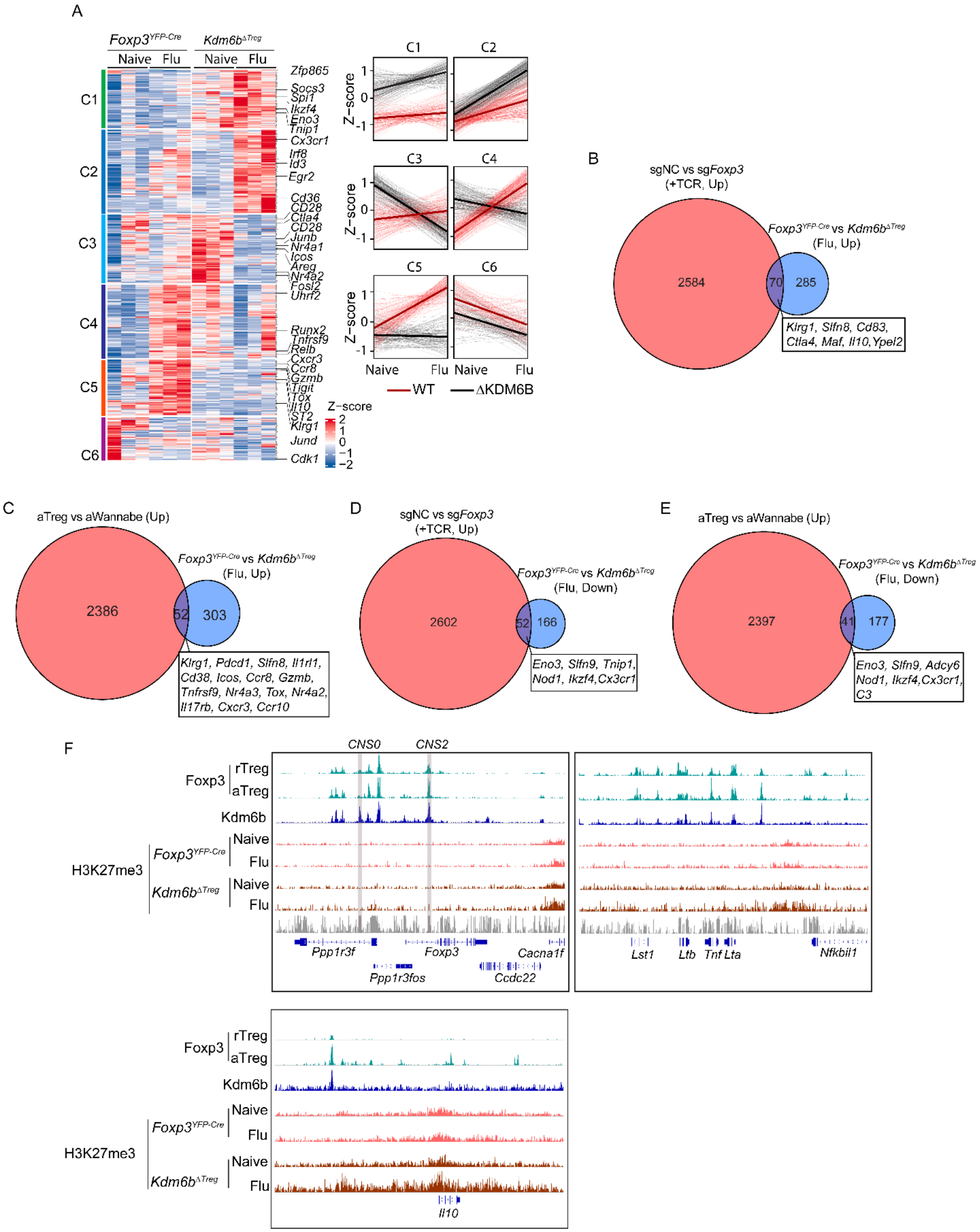


**Supplemental Figure 7. KDM6B impact on lung Treg transcriptome at steady-state and during resolution of lung injury.**

Male and female *Foxp3*$^{YFP\text{-}Cre}$ and *Kdm6b*$^{\Delta Treg}$ mice 8 – 12 weeks of age were examined at either steady state or challenged with intratracheal Influenza A/PR/8/34 H1N1 (PR8) administered at day 0. Mice received 2 μL/g body weight for weight-based dosing as previously described (38). Sorted lung Treg (CD4$^+$YFP$^+$) RNA from either *Foxp3*$^{YFP\text{-}Cre}$ or *Kdm6b*$^{\Delta Treg}$ mice at either steady-state or Day 15 post PR8 influenza were isolated, and RNA-seq was performed. Triplicates per condition.

(**A**) Gene expression patterns in lung Tregs from naive and flu virus-infected *Foxp3*$^{YFP\text{-}Cre}$ or *Kdm6b*$^{\Delta Treg}$ mice. Triplicates per condition.

(**B-E**) Number of genes regulated by FOXP3 (sgNC- versus sgFoxp3-transduced Tregs) (He et al., 2024), or aTreg versus activated Treg wannabe cells (aWannabe) (van der Veeken et al., 2020), and KDM6B in Tregs isolated from flu virus-infected lung.

(**F**) FOXP3, KDM6B, or H3K27me3 peaks at *Foxp3*, *Tnf*, or *IL10* gene regions. FOXP3 or KDM6B CUT&RUN showing binding in aTreg and rTreg cells was overlaid with separate CUT&RUN data showing H3K27me3 modification tracks from sorted in lung Tregs from naive and flu virus-infected *Foxp3*$^{YFP\text{-}Cre}$ or *Kdm6b*$^{\Delta Treg}$ mice. Triplicates per condition.

## Supplemental Table S10

| REAGENT or RESOURCE | SOURCE | IDENTIFIER |
|---|---|---|
| Antibodies | | |
| CD194 (CCR4) anti-mouse - PE/Cy7 (clone: 2G12) | Biolegend | cat# 131214; RRID: AB_2244410 |
| CD103 anti-mouse - BV605 (clone: 2E7) | Biolegend | cat# 121433; RRID: AB_2629724 |
| CD103 anti-mouse - BV785 (clone: 2E7) | Biolegend | cat# 121439; RRID: AB_2800588 |
| CD103 anti-mouse - PE/Cy7 (clone: 2E7) | Biolegend | cat# 121426; RRID: AB_2563691 |
| CD19 anti-mouse - BV605 (clone: 6D5) | Biolegend | cat# 115540; RRID: AB_2563067 |
| CD25 anti-mouse - APC/Cy7 (clone: PC61) | Biolegend | cat# 102026; RRID: AB_830745 |
| CD3 anti-mouse - FITC (clone: 17A2) | Biolegend | cat# 100204; RRID: AB_312661 |
| CD4 anti-mouse - Alexa 700 (clone: GK1.5) | Biolegend | cat# 100430; RRID: AB_493699 |
| CD4 anti-mouse - BV786 (clone: RM4-5) | BD Biosciences | cat# 563727; RRID: AB_2728707 |
| CD44 anti-mouse - BV510 (clone: IM7) | BD Biosciences | cat# 563114; RRID: AB_2738011 |
| CD45 anti-mouse - BV785 (clone: 30-F11) | Biolegend | cat# 103149; RRID: AB_2564590 |
| CD45 anti-mouse - FITC (clone: 30-F11) | Biolegend | cat# 103108; RRID: AB_312973 |
| CD45.2 anti-mouse - PE (clone: 104) | Biolegend | cat# 109808; RRID: AB_313445 |
| CD45.2 anti-mouse - PE/Dazzle (clone: 104) | Biolegend | cat# 109846: RRID: AB_2564177 |
| CD62L anti-mouse - BV650 (clone: MEL-14) | BD Biosciences | cat# 564108; RRID: AB_2738597 |
| CD8α anti-mouse - Alexa 700 (clone: 53-6.7) | Biolegend | cat# 100730; RRID: AB_493703 |
| CD8α anti-mouse - PE/Dazzle 594 (clone: 53-6.7) | Biolegend | cat# 100762; RRID: AB_2564027 |
| Foxp3 anti-mouse - APC (clone: FJK-16s) | Invitrogen | cat# 17-5773-82; RRID: AB_469457 |
| Helios anti-mouse/human - FITC (clone: 22F6) | Biolegend | cat# 137214; RRID: AB_10662745 |
| I-A/I-E (MCH II) anti-mouse - Alexa 700 (clone: M5/114.15.2) | Biolegend | cat# 107622; RRID: AB_493727 |
| IFN-γ anti-mouse - BV510 (clone: XMG1.2) | Biolegend | cat# 505841; RRID: AB_2562187 |
| Influenza A NP Monoclonal-FITC (clone: D67J) | Invitrogen | cat# MA1-7322; RRID: AB_1017747 |
| Ki-67 anti-mouse - BV421 (clone: 16A8) | Biolegend | cat# 652411; RRID: AB_2562663 |
| Ki-67 Monoclonal -PE (clone: SolA15) | Invitrogen | cat# 12-5698-82; RRID: AB_11150954 |
| KLRG1 (MAFA) anti-mouse/human - BV605 (clone: 2F1/KLRG1) | Biolegend | cat# 138419; RRID: AB_2563357 |
| TCR γ/δ anti-mouse - PerCP/Cy 5.5 (clone: GL3) | Biolegend | cat# 118118; RRID: AB_10612756 |
| TNFα anti-mouse - PerCP/Cy5.5 (clone: MP6-XT22) | Biolegend | cat# 506322; RRID: AB_961434 |
| Tri-Methyl-Histone H3 (Lys27) - PE (clone: C36B11) | Cell Signaling Technology | cat# 40724S |
| Tri-Methyl-Histone H3 (Lys27) | Cell Signaling Technology | cat# 9733S |

| | | |
|---|---|---|
| Anti-KDM6B / JMJD3 antibody | Abcam | Cat# ab38113 |
| Zombie Aqua Fixable Viability Kit | Biolegend | cat# 423102 |
| Zombie NIR Fixable Viability Kit | Biolegend | cat# 423106 |
| Chemicals, peptides, and recombinant proteins | | |
| Biolegend cell activation cocktail | Biolegend | Cat# 423304 |
| RPMI-1640 medium | GIBCO, Life Technologies | Cat# 52400-025 |
| Collagenase, type I | Worthington Biochemicals | Cat# LS004197 |
| CFSE | BD Bioscience | Cat# 565082 |
| DNAse | Worthington Biochemicals | Cat# LS002139 |
| Human IL2 | ThermoFisher, Peprotech | Cat# 200-02 |
| Dextran Sulfate Sodium Salt | ThermoFisher, | Cat# J63606.22 |
| Neomycin | Millipore Sigma | Cat# 1458019 |
| Miltenyi CD3/CD28 Bead Kit | Miltenyi | Cat# 130-093-627 |
| Hot Start High-Fidelity DNA Polymerase | New England Biolabs | Cat# M0515S |
| pAG-MNase | He *et al.*[29] | N/A |
| Digitonin | Millipore | Cat# 300410-1GM |
| KAPA Hyper prep kit | Kapa Biosystems | Cat# KK8504 |
| SPRIselect | Beckman Coulter | Cat# B23318 |
| EDTA (0.5 M), pH 8.0, RNase-free | Invitrogen | Cat# AM9261 |
| Low melting agarose | Invitrogen | Cat# 16520100 |
| EGTA | Sigma-Aldrich | Cat# 3889 |
| Percoll | Sigma-Aldrich | Cat# GE17-0891-01 |
| TRIzol RNA isolation reagents | Thermo Fisher Scientific | Cat# 10296010 |
| Mouse T-Activator CD3/CD28 beads | Thermo Fisher Scientific | Cat# 11456D |
| Foxp3 / transcription factor staining buffer set | eBioscience | Cat# 00-5523-00 |
| Spermidine | Sigma-Aldrich | Cat# S2626 |
| Influenza A/PR/8/34 H1N1 (PR8) | Charles River | Cat# 10100374 |
| Critical commercial assays | | |
| EasySep Mouse CD4+CD25+ Regulatory T Cell Isolation Kit II | StemCell Technologies | Cat# 18783 |
| CUT&RUN Assay Kit | Cell Signaling Technologies | Cat# 86652 |
| KAPA Mouse Genotyping Kits - HotStart PCR | Fisher Scientific | Cat# 50-196-5243 |
| Quick-RNA MicroPrep Kit, Zymo Research | Fisher Scientific | Cat# 50-444-592 |
| NucleoSpin Gel and PCR Clean-up Mini Kit | Macherey-Nagel | Cat# 740609.50 |
| Qiagen MinElute PCR Purification Kit | Qiagen | Cat# 28006 |
| DNA Purification Buffer and Spin Column Kit | Cell Signaling Technologies | Cat# 14209 |
| BSA Protein Assay | Bio-Rad Laboratories | Protein Assay Reagent A: 5000113 Protein Assay Reagent B: 5000114 Quick Start BSA Standard 2mg/ml: 5000206 |
| Oligonucleotides | | |
| Kdm6b primers | Sigma | Jmjd3-F: CAGAGGCAGGTAGATCTTTG<br>Jmjd3-F3: GAGGTGAAGAACGTCAAGTC<br>Jmjd3-R: CAACCCTCCCTTTCTTTTCG |
| Foxp3 Cre primers | Sigma | forward: AGGATGTGAGGGACTACCTCCTGTA<br>reverse: TCCTTCACTCTGATTCTGGCAATTT |
| Foxp3 Cre Wildtype primers | Sigma | forward: CCTAGCCCCTAGTTCCAACC<br>reverse: AAGGTTCCAGTGCTGTTGCT |
| Other | | |
| Plasmid pSIR-BbsI-Thy1.1 | Li et al. [31] | |
| | | |
